# A global investigation into antimicrobial knowledge in medicine, pharmacy, nursing, dentistry and veterinary undergraduate students: A scoping review to inform future planetary health multidisciplinary education

**DOI:** 10.1186/s12909-024-06253-w

**Published:** 2024-10-29

**Authors:** Shahd Alzard, Betty Exintaris, Mahbub Sarkar, Averil Grieve, Sara Chuang, Renier Coetzee, Angelina Lim

**Affiliations:** 1 Monash University, Melbourne, VIC 3052 Australia; 2 School of Public Health, University of the Western Cape, Cape Town, South Africa; 3 Murdoch Children’s Research Institute, Royal Children’s Hospital, Parkville, VIC 3052 Australia; 4 Faculty of Pharmacy and Pharmaceutical Sciences, Monash University, Parkville, VIC 3052 Australia

**Keywords:** Antimicrobial prescribing, antimicrobial resistance, antimicrobial stewardship, knowledge, attitude, practice, undergraduate, multidisciplinary, interprofessional education, scoping review

## Abstract

**Background:**

Inappropriate use of antimicrobials can push the environment out of balance, and cause unnecessary waste that can contaminate our soil, animals and waterways. Health professional education is committed to preparing students for antimicrobial stewardship (AMS) and supporting planetary health, but a more multidisciplinary action is needed to curb the expansion of antimicrobial resistance (AMR). The aim of this scoping review is to showcase the current antimicrobial knowledge of undergraduate students across the disciplines of medicine, pharmacy, nursing, dentistry and veterinary. This will consequently showcase the gaps and trends across the different disciplines and countries to help inform planetary health multidisciplinary undergraduate curriculums.

**Methods:**

A search of the existing literature published prior to December 2023 was conducted using CINAHL, EMBASE, MEDLINE, SCOPUS, and ERIC databases. Studies were excluded if they included postgraduate students or discussed the knowledge, attitude and practice (KAP) of students towards antimicrobial prescribing, AMR and AMS related to a specific learning activity.

**Results:**

A total of 144 articles were included. The most represented countries were India and Pakistan accounting for 17% and 8% of the studies, respectively. Single-disciplinary research accounted for approximately 80% of the studies. Medicine was the most represented discipline in both single-disciplinary and multidisciplinary research, appearing in approximately 62% of the studies, followed by pharmacy appearing in approximately 30% of the studies and dentistry appearing in approximately 18% of the studies. Three major priority themes were identified: students are more familiar with the term AMR compared to AMS; inappropriate use of antimicrobials is seen as the main driver of AMR; and the need for more training and education in the field of appropriate antimicrobial prescribing, AMR and AMS.

**Conclusion:**

This review has highlighted that there is a need for more AMS interprofessional education (IPE) activities in all five disciplines, and especially within the disciplines of nursing, veterinary and dentistry, as shown by a lack of multidisciplinary research in this area. Most of the knowledge assessments have just touched the surface of AMS and focused on inappropriate antimicrobial use alone. Interdisciplinary planetary health education needs to go beyond these skills and broaden the understanding of other factors that can contribute to AMR such as inappropriate disposal, environmental contamination, monitoring and surveillance, one health, false allergies, and more importantly, how each health professional can contribute to a team.

**Supplementary Information:**

The online version contains supplementary material available at 10.1186/s12909-024-06253-w.

## Background

Antimicrobial drugs have been heavily misused in humans, animals and environmental settings leading to the emergence of antimicrobial resistance (AMR), which can jeopardise the health of the planet [1, 2]. Antimicrobials utilized in both human and veterinary medicine for treating or preventing infections can find their way into waterways through emissions from pharmaceutical manufacturing, agricultural runoff, biological excretion from humans and animals, and the disposal of unused medicines [1, 2]. In aquatic environments, antibiotic residues at sub-lethal levels create selective pressure that enhances AMR. Antimicrobial-resistant genes can be transferred between different species and ecosystems. Consequently, waterways can become sources of antimicrobial-resistant pathogens that infect humans and animals, leading to increased antimicrobial use and potentially worsening AMR [1, 2].

Health professionals have a vital role in supporting planetary health and reducing the risk of AMR. Education prior to practice is an essential step to equip health professionals with the appropriate knowledge to optimize antimicrobial use. The World Health Organisation (WHO) has deemed AMR as one of the top 10 global public health threats facing humanity [3], advocating worldwide for the development and implementation of antimicrobial stewardship (AMS) programs, as the AMS and AMR education of health professionals is of utmost importance [4]. AMS refers to the responsible, safe and judicious use and prescribing of antimicrobial drugs, and aims to mitigate the burden and public health impact of AMR [5]. Moreover, optimal outcomes are best achieved when improving AMS is conducted in an interprofessional manner through a collaborative effort between health professionals from different disciplines [6, 7].

Implementing AMS education early on in the undergraduate curriculum [8, 9], at a time when the knowledge and attitudes of health professionals are still in the process of being shaped rather than changed, will increase the likelihood of retention of knowledge and clinical implementation post-graduation [10, 11]. Considering its importance and expanding significance in diverse practice settings, the aim of this scoping review is to showcase the knowledge, attitude and practice (KAP) of undergraduate students towards antimicrobial prescribing, AMR and AMS across the disciplines of medicine, pharmacy, nursing, dentistry and veterinary. This will consequently showcase the gaps and trends across different disciplines and countries to help inform planetary health multidisciplinary undergraduate curriculums. It is hoped that this review will provide a strong foundation for health professional academics to instigate interprofessional AMS innovations and programs.

## Methods

A scoping review methodology was chosen as it provided the most effective way to fully explore the existing literature on the KAP towards antimicrobial prescribing, AMR and AMS in the undergraduate curricula of the disciplines of medicine, pharmacy, nursing, dentistry and veterinary, and to identify and analyse knowledge gaps. A systematic search was conducted in CINAHL, EMBASE, MEDLINE, SCOPUS, and ERIC databases to identify relevant studies published prior to December 2023. The Joanna Briggs Institutes (JBI) manual framework for scoping reviews was followed when conducting this scoping review [12]. The scoping review process consisted of five main steps. Firstly, the research question was identified. The second step involved a comprehensive search strategy to identify relevant studies. The third step involved the selection of relevant studies according to the predetermined inclusion and exclusion criteria. The fourth step was to extract the data and chart the details of the included studies. The fifth stage involved the analysis and presentation of the study findings.

### Identification of relevant studies

After defining the research question, a preliminary literature search was completed to identify a set of ‘gold’ articles [13] that could be used in the review. Following this, a search strategy was conducted on CINAHL, EMBASE, MEDLINE, SCOPUS, and ERIC databases to identify relevant studies in consultation with a specialist health librarian. A manual search of the references cited in the included studies was also undertaken to ensure inclusion of all relevant studies. Moreover, the gold articles were searched within the database to ensure that they were also included. The search strategy included all identified keywords, Medical Subject Headings (MeSH), index terms and text words contained in titles and abstracts of relevant articles. As an example, the search strategy adapted for the MEDLINE database via the OVID platform is found in Table 1.

**Table 1:** Search strategy on OVID Medline

Search	Query	Records retrieved
1	Students, Pharmacy/	4,558
2	Students, Medical/	44,196
3	Students, Nursing/	30,902
4	Students, Dental/	7,329
5	Education, Veterinary/	5,640
6	((medic* or pharmac* or nursing or vet* or dent*) adj4 (undergraduate* or student*)).ti, ab.	90,688
7	1 or 2 or 3 or 4 or 5 or 6	126,469
8	Knowledge/	14,700
9	Attitude/	54,301
10	belief*.ti, ab.	88,802
11	Perception/	43,905
12	view*.ti, ab.	469,813
13	knowledge.ti, ab.	749,895
14	attitude*.ti, ab.	161,909
15	perception*.ti, ab.	278,152
16	8 or 9 or 10 or 11 or 12 or 13 or 14 or 15	1,591,930
17	Antimicrobial Stewardship/	3,451
18	exp Drug Resistance, Microbial/	184,133
19	Communicable Diseases/	33,987
20	XXXntimicrobe*.ti, ab.	181,668
21	Anti-Bacterial Agents/	405,501
22	antibiotic*.ti, ab.	356,033
23	(drug resistance adj3 microbial).ti, ab.	166
24	17 or 18 or 19 or 20 or 21 or 22 or 23	766,902
25	7 and 16 and 24	275

The 275 retrieved records were uploaded onto EndNote, and 148 duplicated records were removed. The remaining 127 records were then imported into Covidence.

### Selection of relevant and reliable studies

Following the search, all identified citations from the different databases were collated and uploaded into EndNote 20 bibliographic software (Clarivate Analytics, PA, USA) and the duplicate records were removed. The remaining search results were then imported to Covidence online software (Veritas Health Innovation, Melbourne, Australia) [14]. More duplicates were then removed either automatically by Covidence or manually by the reviewers. The titles and abstracts were then screened for eligibility by two reviewers (SA and SC) for assessment against the eligibility inclusion and exclusion criteria for the review. A meeting was then conducted with the team before the two reviewers (SA and SC) independently performed full-text screening against the eligibility inclusion and exclusion criteria which is found in Table 2. Conflicts were managed by a third reviewer (AL), and resolved through discussion with the team (SA, SC and AL).

**Table 2:** Inclusion and exclusion criteria of the scoping review

Criterion	Inclusion	Exclusion
Language	English	Non-English
Type of article	Original research, peer-reviewed articles	Not original research, not peer-reviewed, commentaries, editorials, letters, conference abstracts
Study focus	KAP of undergraduate students about antimicrobial prescribing, antimicrobial resistance, antimicrobial stewardship	No mention of KAP, articles that mention a form of learning (games, simulations, interventions etc.) as a comparative mean to show changes in students’ perspectives
Participants	Medicine, pharmacy, nursing, dentistry and veterinary undergraduate students	Medicine, pharmacy, nursing, dentistry and veterinary postgraduate students and healthcare professionals in the workforce
Geographical area of interest	Any country	Nil

**Fig. 1:**
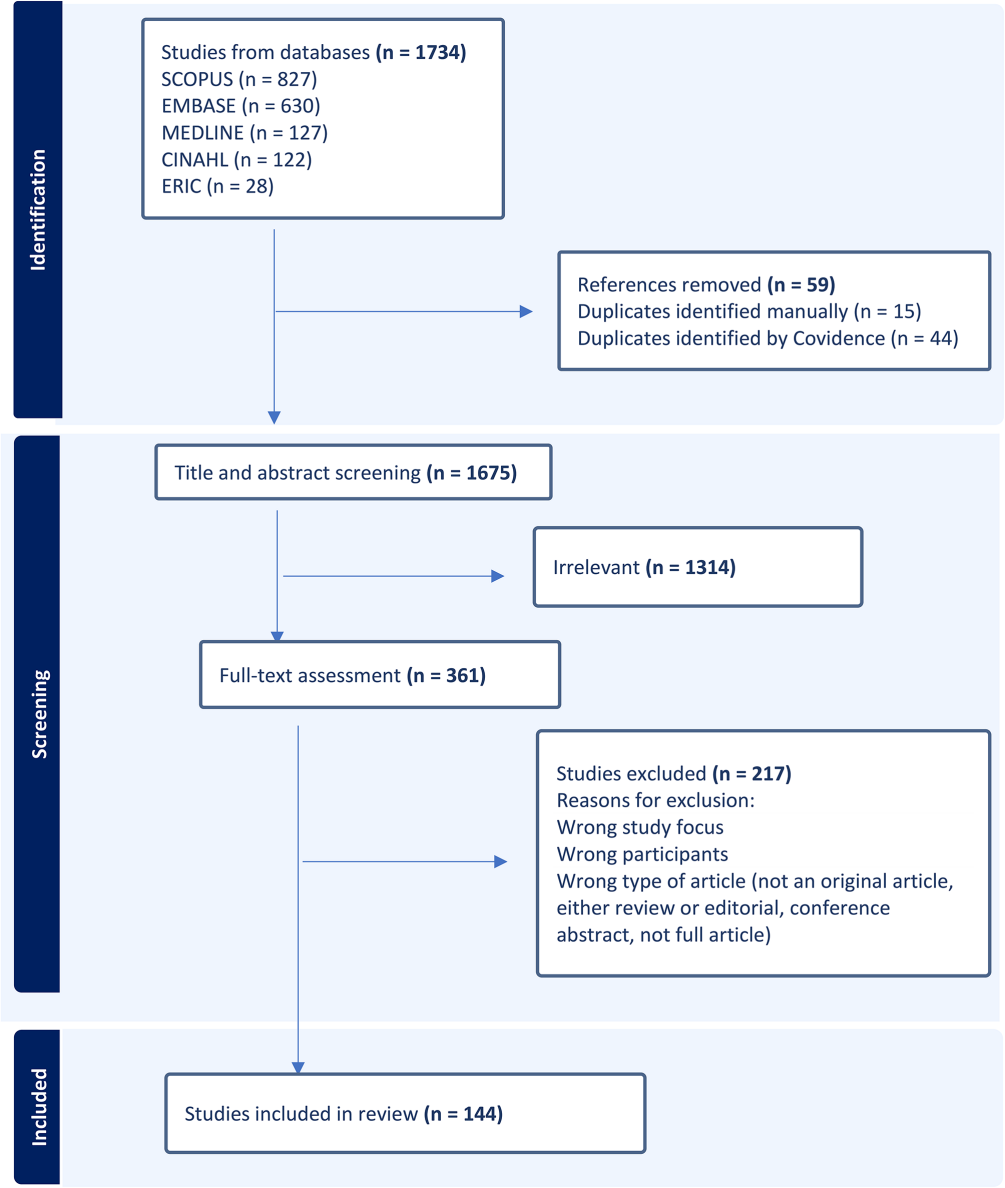
PRISMA flow diagram illustrating the process for selecting articles for scoping review

As shown in the Preferred Reporting Items for Systematic Reviews and Meta-Analyses (PRISMA) flow diagram below (Fig. 1), a total of 1734 records were identified from the search which were then imported to Covidence. After removing duplicates (59 records) and screening the titles and abstracts of 1675 records, 1314 were deemed irrelevant to be included in the review. A total of 361 records then progressed to full-text screening, followed by excluding 217 records. After applying eligibility criteria to the full texts, 144 articles were determined to be eligible for inclusion in the review based on the inclusion/exclusion eligibility criteria.

### Data extraction and data analysis

In line with the guidelines for reporting scoping reviews and the JBI framework, the fourth stage involved tabulating details of the included relevant studies and extracting the data. This was then followed by the fifth and final stage of the scoping review which involved analysing the data extracted and presenting study findings. The results were then summarised and reported to identify implications of the study findings for practice and future scholarly research. In addition, content analysis of the main findings of each study was conducted to elicit main themes across the studies. Content analysis was conducted by two authors (SA and SC) with regular meetings with author AL.

## Results

### Characteristics of the included studies

This review yielded 144 articles as shown in Appendix – Table 1. As shown in Appendix – Table 2, all studies were published in the last 10 years between 2013 and 2023, except for three studies that were published in 2004, 2010 and 2011 [15–17]. As shown in Appendix – Table 3, a diverse number of countries were represented in the studies, with the most represented countries being India (approximately 17% of the studies) and Pakistan (approximately 8% of the studies). As shown in Appendix – Table 4, the study type of the majority of the studies was quantitative and employed a cross-sectional survey/questionnaire as the study design for data collection. Four studies followed a mixed-methods (quantitative and qualitative) approach [18–21], and two studies followed a qualitative approach [22, 23]. Qualitative studies primarily employed focus groups and/or semi-structured interviews to capture data.

The articles included single-disciplinary and multidisciplinary research. As shown in Appendix – Table 5, the majority of the studies focused on one discipline at a time where the survey questions mainly concentrated on the domains of knowledge and practice of medicine, pharmacy, nursing, dentistry or veterinary students. Single-disciplinary research accounted for approximately 80% of the studies. Multidisciplinary research, that focused on more than one discipline at a time, was represented in the remaining studies. Only one of the studies represented all five disciplines [24]. As shown in Appendix – Table 6, medicine was the most represented discipline in both single-disciplinary and multidisciplinary research (the majority of the articles being published from India), appearing in approximately 62% of the studies, followed by pharmacy appearing in approximately 30% of the studies and dentistry appearing in approximately 18% of the studies. Regarding the overall number of respondents that participated, the sample size in the studies ranged from 18 [22] to 7328 [24].

The data collated from the studies can be grouped into three major priority themes: There is higher familiarity with the term AMR compared to AMS; Inappropriate use of antimicrobials is seen as the main driver of AMR; and Undergraduate students highlight the need for more education and training in appropriate antimicrobial prescribing, AMR and AMS.

#### Theme 1: There is higher familiarity with the term AMR compared to AMS

Considering that AMR is a major global health challenge, the education and training of students as part of AMS is vital in improving antimicrobial prescribing patterns in order to minimise the risk of AMR. As shown in Appendix – Table 7, amongst the studies that reported on students’ familiarity with AMS, only approximately 34.2% of them reported that more than 50% of the students surveyed were familiar with AMS and the remaining 65.8% of the studies reported that less than 50% of the students were familiar with AMS.

In contrast, there were more studies that reported on students’ familiarity with AMR compared to AMS. As shown in Appendix – Table 8, amongst the studies that reported on students’ familiarity with AMR, approximately 90.1% of the studies reported that more than 50% of the students surveyed were familiar with AMR. Therefore, there were significantly more students familiar with AMR compared to AMS. Moreover, approximately 38.5% of the studies reported that more than 90% of students were familiar with AMR and only approximately 7.9% of the studies reported that more than 90% of students were familiar with AMS. Generally, it has also been noted that a high familiarity with AMS correlates with a high familiarity with AMR, but the reverse is not necessarily true.

Data was also further organised to isolate responses regarding the familiarity with AMR and AMS on the basis of disciplines. It was found that medicine and pharmacy were the uppermost disciplines that were familiar with AMS and AMR. As shown in Appendix – Table 9, an average of approximately 35.5% and 79.4% of the medical students were familiar with AMS and AMR, respectively. Whereas, an average of approximately 50.2% and 79.2% of the pharmacy students were familiar with AMS and AMR, respectively.

#### Theme 2: Inappropriate use of antimicrobials is seen as the main driver of AMR

The most important factor that can contribute to AMR reported in the studies is the inappropriate use of antimicrobials, which includes misusing antimicrobials and unnecessary use of broad-spectrum antimicrobials when equally effective narrow-spectrum antimicrobials can be used instead. Similar to theme 1, medicine and pharmacy were, overall, the disciplines that had the highest number of students reporting on the factors contributing to AMR. As shown in Appendix – Table 10, an average of approximately 82% and 67.4% of the medical students believed that the main factors contributing to AMR were the inappropriate use of antimicrobials and the unnecessary use of broad-spectrum antimicrobials to treat infections, respectively. Whereas, an average of approximately 80.4% and 73.8% of the pharmacy students believed that the main factors contributing to AMR were the inappropriate use of antimicrobials and the unnecessary use of broad-spectrum antimicrobials to treat infections, respectively.

Additionally, a factor that can also contribute to AMR is the use of antibiotics for incorrect indications. As shown in Appendix – Table 13, studies also reported that a high proportion of the students surveyed agreed that antibiotics were mistakenly prescribed for incorrect indications (such as common cold and/or viral infections), that antibiotics are ineffective against viruses, and/or that bacteria were not responsible for common cold/flu.

In this theme, the highest number of responses regarding the knowledge of antimicrobial prescribing was from medical students. As shown in Appendix – Table 11, the data showed that an average of approximately 85.7%, 68.6%, 48.6% and 82.5% of the medical students knew when to initiate antimicrobial treatment, how to select the most appropriate antimicrobial for the infection, how to choose an appropriate dosage regimen of antimicrobials (e.g. dose, dose frequency, route of administration, duration of treatment), and when to de-escalate from intravenous to oral route, respectively.

Regarding the discipline of dentistry, most of the studies reported that dental students may generally prescribe antibiotics inappropriately to manage conditions when not indicated, such as periapical abscess, dry socket and pulpitis. Moreover, most of the students chose to prescribe amoxicillin as their first-choice of antibiotic for such infections [20, 25–28].

#### Theme 3: Undergraduate students highlight the need for more education and training in appropriate antimicrobial prescribing, AMR and AMS. 

In addition to recognising that a comprehensive knowledge of antimicrobials, AMR and AMS is important in their careers, students in the studies covered in this review also highlighted the need for further education/training on appropriate antimicrobial use/AMR/AMS. Similar to the previous themes, data was also further analysed to isolate students’ responses on the basis of disciplines. In this theme, the highest number of responses was from medical students. As shown in Appendix – Table 12, the data showed that an average of approximately of 86.4%, 83%, 83% and 69% of the medical students acknowledged the importance of knowledge of antimicrobials/AMR/AMS in their careers, highlighted the need for further education/training on appropriate antimicrobial use, AMR and AMS, respectively. The second highest number of responses in this theme was from pharmacy students. The data showed that an average of approximately of 95.4%, 92.7%, 91% and 90.7% of pharmacy students acknowledged the importance of knowledge of antimicrobials/AMR/AMS in their careers, highlighted the need for further education/training on appropriate antimicrobial use, AMR and AMS, respectively.

## Discussion

This scoping review included 144 studies on the KAP of undergraduate students towards antimicrobial prescribing, AMR and AMS from the disciplines of medicine, pharmacy, nursing, dentistry and veterinary. Considering the significance of multidisciplinary collaboration and the appreciation of the unique skills that each healthcare professional brings to improve health outcomes, patient care and achieve optimal AMS, this review focused on the five health disciplines. The top publishing countries in this review were found to be India and Pakistan. This could be attributed to the fact that research is deemed necessary in areas where antimicrobials are misused, thus contributing to a very high rate of AMR in these countries. In India and Pakistan where there are insufficient pharmaceutical regulations of antimicrobials, antibiotics are readily available over-the-counter in local pharmacies and without a prescription [29–32]. This can result in patients self-treating with antibiotics and possibly for conditions that do not require antibiotic treatment [33]. Given the fundamental role of prescribing and facilitating the supply of antimicrobial agents, doctors and pharmacists have a greater potential in curbing the expansion of AMR [34, 35]. Therefore, it is understandable why interest is taken in surveying these disciplines, thus, resulting in more representation in the survey data as shown in the aforementioned results.

Medical doctors work across different settings where they need to accurately diagnose and treat infections which tends to include prescribing antimicrobials. It is the responsibility of all doctors to use evidence-based guidelines and resources in their practice of prescribing, and to follow safe and rational prescribing and AMS principles in order to minimise the risk of AMR [36–38]. Pharmacists play multiple roles that can minimise the expansion of AMR and promote AMS, including optimising antimicrobial therapy, recommending an appropriate dosage regimen and duration of therapy, therapeutic drug monitoring and instructing patients on the appropriate use of antimicrobials and their potential adverse effects [39–43].

The disciplines of nursing, dentistry and veterinary were generally the least represented in the survey data across the themes. This is unfortunate considering the important role that nurses, dentists and veterinarians can play in optimal antimicrobial prescribing, curbing AMR and promoting AMS. All human and animal healthcare professionals are equally responsible for minimising the risk of AMR and promoting AMS [24]. For instance, nurses can play a key role in minimising the risk of AMR by obtaining cultures before antimicrobials are administered, prompting de-escalation of therapy when possible, monitoring for patient safety, promoting optimal antibiotic use and providing patient and staff education [18, 44, 45]. Given that they are prescribers of antimicrobials for dental purposes, dentists should also be responsible for appropriate antimicrobial prescribing. As shown in theme 2, a high proportion of dentists prescribe antimicrobials unnecessarily and when not indicated. It has also been suggested that as much as 80% of antibiotic prescribing by dentists may be inappropriate [46–48]. Given the concerning emergence and spread of bacteria resistant to antibiotics in animals, veterinarians also ought to play a role in appropriate antimicrobial prescribing and curbing AMR [49, 50]. Therefore, in order to ensure that healthcare professionals from different disciplines are encouraged to partake in AMS programs, more studies need to be undertaken to involve all of the five healthcare disciplines.

From the results obtained in theme 1 regarding the familiarity of students with AMS and AMR, it was found that students’ familiarity with AMS was lower than that with AMR. This may be due to less attention being paid to delivering AMS education or training programs to students [16, 51–54], and more focus being on the overall detrimental effects of AMR to the healthcare system. This is also further evidenced by the WHO and the vast number of studies which have reinforced the importance of implementing AMS education/training programs in the curricula as an integral part of AMR containment activities [55–57]. However, there could be certain barriers to implementing AMS programs that need to be taken into consideration, such as staffing constraints, remuneration, lack of funding and lack of administrative support [58–60]. Considering that the term ‘AMS’ does not have a specifically clear universal definition, it can also be difficult to classify what is and what is not AMS. Nonetheless, AMS knowledge has definitely expanded in recent years. Dyar et al. reported that results for the search term ‘(antimicrobial OR antibiotic) AND stewardship’ were over 2500, due to its exponential use in the last five years [50]. AMS would be further enhanced through multidisciplinary teams and multidisciplinary research and this review shows a lack of multidisciplinary research in this area.

From the results obtained in theme 2, the main factors contributing to AMR were found to be the inappropriate use of antimicrobials. This may be due to a lack of tertiary education regarding the appropriate use of antimicrobials, which is also part of effective AMS education [61]. AMS education should include information about minimising the unnecessary use of antimicrobials and utilising susceptibility/microbiology test results to guide directed therapy to promote the use of narrow-spectrum antimicrobials [62]. Knowing the causative microbial agent encourages the use of narrow-spectrum antimicrobial agents that act against specific species, preferably over broad-spectrum antimicrobials. Whilst they are still important as a form of initial empirical therapy, particularly in cases where delayed treatment can result in harmful health effects, broad-spectrum antimicrobials can increase the spread of AMR and have detrimental effects on the host microbiome especially if used for prolonged durations [63, 64]. Therefore, educating students and clinicians about the use and interpretation of microbiological investigations to guide therapy should definitely be encouraged as part of AMS interventions.

Similarly, and as seen from the results obtained in theme 3, the principles of AMS education should also include elements of prudent antimicrobial use such as knowing when to initiate antimicrobial therapy, ensuring that the right antimicrobial is used for the correct infection and choosing a correct dosage regimen (e.g. dose, dose frequency, route, duration of treatment) [50, 65]. Moreover, education should also involve patient monitoring and knowing when to de-escalate from IV to oral therapy when the patient is deemed clinically stable [42]. Further research is encouraged to develop a standardised definition of certain AMS principles such as both appropriate and inappropriate antimicrobial use, time to administration of appropriate therapy, duration of therapy and adverse reactions related to antimicrobial therapy [65]. Apart from prescribing, other factors that can contribute to AMR such as inappropriate disposal, environmental contamination, monitoring and surveillance, one health, false allergies were not widely assessed and represents an area that could be targeted to help multidisciplinary teams think broader than just prescribing alone.

From the results obtained in theme 3, a vast majority of respondents surveyed in the studies included in this review have expressed interest in further education/training on appropriate antimicrobial use, AMR and AMS. The implementation of AMS education and training early in the undergraduate curricula is highly encouraged [16, 61, 66]. Such implementation at a time when the knowledge, attitude and perceptions of students are still being shaped is believed to yield highly successful results [67]. When students, however, graduate and go into their professional workforce, it can be challenging to change their views and behaviours [68]. Institutions across Australia, the UK and USA deliver AMS teachings to undergraduate students in different forms such as didactic lectures, case studies, problem-based learning modules, e-learning resources, journal clubs, and objective structured clinical examinations (OSCEs) on AMR, infectious diseases, antibiotic prescribing, hygiene and infection control, and AMS principles [10, 66, 69, 70].

Moreover, optimal AMS outcomes can be achieved via a multidisciplinary interprofessional approach where healthcare professionals from different disciplines work and collaborate together towards minimising the risk of AMR and promoting AMS programs [50, 71]. By being part of multidisciplinary teams, both human and animal healthcare professionals are able to bring their individual expertise together to better address complex global health issues such as AMR and AMS [24, 53]. AMS interprofessional education (IPE) activities in students could be designed from AMS IPE activities that have been delivered to healthcare professionals, and could cover topics such as necessary use of antibiotics and antifungals, adverse effects and AMR, AMS concepts, surveillance, infection control and how to facilitate AMS interventions together as a multidisciplinary team [72].

### Strengths and limitations

Consistent with the scoping review methodology, the literature was extensively scoped and mapped across a range of study designs in the area of KAP of undergraduate students towards antimicrobial prescribing, resistance and stewardship. This review included the five disciplines of medicine, pharmacy, nursing, dentistry and veterinary. As degrees from different institutions vary across the globe, a comparison made within similar levels of curriculum was not done. Another limitation is that grey literature was not included due to duplication concerns for when abstracts of the preliminary findings were published.

## Conclusion

This review has highlighted that there is a need for more AMS IPE activities in all five disciplines, and especially within the disciplines of nursing, veterinary and dentistry, as shown by a lack of multidisciplinary research in this area. Most of the knowledge assessments have just touched the surface of AMS and focused on inappropriate antimicrobial use alone. Interdisciplinary planetary health education needs to go beyond these skills and broaden the understanding of other factors that can contribute to AMR such as inappropriate disposal, environmental contamination, monitoring and surveillance, one health, false allergies, and more importantly, how each healthcare professional can contribute to a team.

## Electronic Supplementary Material

Below is the link to the electronic supplementary material.

Supplementary Material 1

## Data Availability

All data generated or analysed during this study are included in this published article [and its supplementary information files].

## Abbreviations

AMS: Antimicrobial Stewardship
KAP: Knowledge, Attitude and Practice
AMR: Antimicrobial Resistance
WHO: World Health Organisation
JBI: Joanna Briggs Institutes
MeSH: Medical Subject Headings
PRISMA: Preferred Reporting Items for Systematic Reviews and Meta–Analyses
OSCE: Objective Structured Clinical Examination
IPE: Interprofessional Education

## References

[1] Danner MC, et al. <ArticleTitle Language=“En”>Antibiotic pollution in surface fresh waters: occurrence and effects. Sci Total Environ. 2019;664:793–804. 30763859 10.1016/j.scitotenv.2019.01.406

[2] Ellabaan MMH, et al. Forecasting the dissemination of antibiotic resistance genes across bacterial genomes. Nat Commun. 2021;12(1):2435. 33893312 10.1038/s41467-021-22757-1 PMC8065159

[3] Walsh TR, et al. Antimicrobial Resistance: Addressing a Global Threat to Humanity. PLoS Med. 2023;20(7):e1004264. 37399216 10.1371/journal.pmed.1004264 PMC10317217

[4] World Health Organisation. Promoting antimicrobial stewardship to tackle antimicrobial resistance. 2021 January 24, 2024]; https://www.who.int/europe/activities/promoting-antimicrobial-stewardship-to-tackle-antimicrobial-resistance

[5] Lanckohr C, Bracht H. Antimicrobial stewardship. Curr Opin Crit Care. 2022;28(5):551–6. 35942707 10.1097/MCC.0000000000000967

[6] Przymuszała P, et al. Factors influencing behavioral intentions of graduating pharmacy students regarding interprofessional collaboration - a theory-driven qualitative study. BMC Health Serv Res. 2023;23(1):1207. 37926826 10.1186/s12913-023-10224-0 PMC10626734

[7] World Health Organisation. Framework for Action on Interprofessional Education & Collaborative Practice. 2010 January 26, 2024]; https://www.who.int/publications/i/item/framework-for-action-on-interprofessional-education-collaborative-practice

[8] Alsaleh N, et al. Medical and dental students’ knowledge and perceptions about antimicrobial stewardship: a call for educational enhancement. Military Med Sci Lett (Vojenske Zdravotnicke Listy). 2020;89(4):207–14.

[9] Patel N, Begum S, Kayyali R. Interprofessional Education (IPE) and Pharmacy in the UK. A Study on IPE Activities across Different Schools of Pharmacy. Pharm (Basel), 2016. 4(4). 10.3390/pharmacy4040028 PMC5419369 28970401

[10] Pulcini C, Gyssens IC. How to educate prescribers in antimicrobial stewardship practices. Virulence. 2013;4(2):192–202. 23361336 10.4161/viru.23706 PMC3654620

[11] Silverberg SL, et al. A review of antimicrobial stewardship training in medical education. Int J Med Educ. 2017;8:353–74. 29035872 10.5116/ijme.59ba.2d47 PMC5694692

[12] Peters MD et al. *JBI manual for evidence synthesis.* JBI Manual for Evidence Synthesis, 2020: pp. 406–451.

[13] LIBRARY MU. *Systematic Review: Review & test your search*. 2024 24/06/2024]; https://guides.lib.monash.edu/systematic-review/review-and-test-your-search

[14] Covidence software. Veritas Health Innovation, Melbourne, Australia.

[15] Wright E, Jain P. Survey of antibiotic knowledge amongst final year medical students. J Antimicrob Chemother. 2004;53(3):550–1. 14762052 10.1093/jac/dkh096

[16] Minen MT, et al. A survey of knowledge, attitudes, and beliefs of medical students concerning antimicrobial use and resistance. Microb Drug Resist. 2010;16(4):285–9. 20624097 10.1089/mdr.2010.0009

[17] Bp S et al. Survey on knowledge towards antibiotics among the nursing students. Int J Pharm Pharm Sci, 2011. 3.

[18] Bouchoucha SL, et al. Nursing students’ awareness and perceptions of nurses’ role in antimicrobial stewardship. Nurse Educ Pract. 2021;52:103036. 33836385 10.1016/j.nepr.2021.103036

[19] Khan FU, et al. Exploring Undergraduate Pharmacy Students Perspectives Towards Antibiotics Use, Antibiotic Resistance, and Antibiotic Stewardship Programs Along With the Pharmacy Teachers’ Perspectives: A Mixed-Methods Study From Pakistan. Front Pharmacol. 2021;12:754000. 34819859 10.3389/fphar.2021.754000 PMC8606649

[20] Schneider-Smith EG, et al. How decisions are made: Antibiotic stewardship in dentistry. Infect Control Hosp Epidemiol. 2023;44(11):1731–6. 37553682 10.1017/ice.2023.173 PMC10782556

[21] Wiese-Posselt M, et al. Appropriate antibiotic use and antimicrobial resistance: knowledge, attitudes and behaviour of medical students and their needs and preferences for learning. Antimicrob Resist Infect Control. 2023;12(1):48. 37198699 10.1186/s13756-023-01251-x PMC10189209

[22] Primeau CA, et al. Exploring medical and veterinary student perceptions and communication preferences related to antimicrobial resistance in Ontario, Canada using qualitative methods. BMC Public Health. 2023;23(1):483. 36915074 10.1186/s12889-023-15193-x PMC10012462

[23] Vázquez-Lago JM et al. Knowledge, Perceptions, and Perspectives of Medical Students Regarding the Use of Antibiotics and Antibiotic Resistance: A Qualitative Research in Galicia, Spain. Antibiot (Basel), 2023. 12(3). 10.3390/antibiotics12030558 PMC10044116 36978424

[24] Dyar OJ et al. Assessing the Knowledge, Attitudes and Behaviors of Human and Animal Health Students towards Antibiotic Use and Resistance: A Pilot Cross-Sectional Study in the UK. Antibiot (Basel), 2018. 7(1). 10.3390/antibiotics7010010 PMC5872121 29385687

[25] AboAlSamh A, et al. Dental Students’ Knowledge and Attitudes towards Antibiotic Prescribing Guidelines in Riyadh, Saudi Arabia. Volume 6. Pharmacy (Basel); 2018. 2. 10.3390/pharmacy6020042 PMC6025366 29735914

[26] Anandakumar S, Sankari R. Knowledge, attitude and practice on antibiotic therapy among dental students-a pilot study. Res J Pharm Technol. 2018;11(6):2473–5.

[27] Hanweet DM, Mahdi KA, Ahmed AQ. Knowledge and Attitude of Antibiotic Prescription Among Dental Students in Najaf City, Iraq. Int Neurourol J. 2023;27(4):1387–91.

[28] Indrapriyadharshini K et al. Knowledge about antibiotic resistance among dental students in Chengalpattu district, Tamil Nadu – A cross-sectional study. J Global Oral Health, 2021. 4.

[29] Chokshi A et al. Global Contributors to Antibiotic Resistance. J Global Infect Dis, 2019. 11(1). 10.4103/jgid.jgid_110_18 PMC6380099 30814834

[30] Kotwani A, Joshi J, Lamkang AS. Over-the-Counter Sale of Antibiotics in India: A Qualitative Study of Providers’ Perspectives across Two States. Antibiot (Basel), 2021. 10(9). 10.3390/antibiotics10091123 PMC8472180 34572705

[31] Majid Aziz M et al. Dispensing of Non-Prescribed Antibiotics from Community Pharmacies of Pakistan: A Cross-Sectional Survey of Pharmacy Staff’s Opinion. Antibiot (Basel), 2021. 10(5). 10.3390/antibiotics10050482 PMC8143445 33922058

[32] Okeke IN, Lamikanra A, Edelman R. Socioeconomic and behavioral factors leading to acquired bacterial resistance to antibiotics in developing countries. Emerg Infect Dis. 1999;5(1):18–27. 10081668 10.3201/eid0501.990103 PMC2627681

[33] Dadgostar P. Antimicrobial Resistance: Implications and Costs. Infect Drug Resist. 2019;12:3903–10. 31908502 10.2147/IDR.S234610 PMC6929930

[34] Charani E, Cooke J, Holmes A. Antibiotic stewardship programmes—what’s missing? J Antimicrob Chemother. 2010;65(11):2275–7. 20851812 10.1093/jac/dkq357

[35] Rusic D, et al. Antimicrobial resistance: physicians’ and pharmacists’ perspective. Microb Drug Resist. 2021;27(5):670–7. 33052767 10.1089/mdr.2020.0272

[36] Australian Commission on Safety and Quality in Health Care. Role of prescribers in antimicrobial stewardship. 2018 08/03/2024]; https://www.safetyandquality.gov.au/sites/default/files/migrated/Chapter10-Role-of-prescribers-in-antimicrobial-stewardship.pdf

[37] Simpson SA, Wood F, Butler CC. General practitioners’ perceptions of antimicrobial resistance: a qualitative study. J Antimicrob Chemother. 2006;59(2):292–6. 17110392 10.1093/jac/dkl467

[38] Zgliczyński WS, Bartosiński J, Rostkowska OM. Knowledge and Practice of Antibiotic Management and Prudent Prescribing among Polish Medical Doctors. Int J Environ Res Public Health, 2022. 19(6). 10.3390/ijerph19063739 PMC8954040 35329427

[39] American Journal of Health-System Pharmacy. ASHP Statement on the Pharmacist’s Role in Antimicrobial Stewardship and Infection Prevention and Control. Am J Health-System Pharm. 2010;67(7):575–7. 10.2146/sp100001 20237387

[40] Australian Commission on Safety and Quality in Health Care. Role of the pharmacist and pharmacy services in antimicrobial stewardship. 2018 08/03/2024]; https://www.safetyandquality.gov.au/sites/default/files/migrated/Chapter11-Role-of-the-pharmacist-and-pharmacy-services-in-antimicrobial-stewardship.pdf

[41] Dyar OJ, Tebano G, Pulcini C. Managing responsible antimicrobial use: perspectives across the healthcare system. Clin Microbiol Infect. 2017;23(7):441–7. 28433726 10.1016/j.cmi.2017.04.016

[42] Garau J, Bassetti M. Role of pharmacists in antimicrobial stewardship programmes. Int J Clin Pharm. 2018;40(5):948–52. 30242589 10.1007/s11096-018-0675-z

[43] Lai WM et al. Pharmacists’ Perspectives of Their Roles in Antimicrobial Stewardship: A Qualitative Study among Hospital Pharmacists in Malaysia. Antibiot (Basel), 2022. 11(2). 10.3390/antibiotics11020219 PMC8868356 35203822

[44] Edwards R, et al. Covering more Territory to Fight Resistance: Considering Nurses’ Role in Antimicrobial Stewardship. J Infect Prev. 2011;12(1):6–10. 21532974 10.1177/1757177410389627 PMC3083718

[45] Rábano-Blanco A et al. Nursing Students’ Knowledge and Awareness of Antibiotic Use, Resistance and Stewardship: A Descriptive Cross-Sectional Study. Antibiot (Basel), 2019. 8(4). 10.3390/antibiotics8040203 PMC6963445 31671525

[46] Cooper L, et al. Tackling antimicrobial resistance in practice: dental students’ evaluation of university teaching supplemented by an online course. JAC Antimicrob Resist. 2022;4(2):dlac039. 35415610 10.1093/jacamr/dlac039 PMC8994195

[47] Suda KJ, et al. Assessment of the Appropriateness of Antibiotic Prescriptions for Infection Prophylaxis Before Dental Procedures, 2011 to 2015. JAMA Netw Open. 2019;2(5):e193909–193909. 31150071 10.1001/jamanetworkopen.2019.3909 PMC6547109

[48] Teoh L, Thompson W, Suda K. Antimicrobial stewardship in dental practice. J Am Dent Association. 2020;151(8):589–95. 10.1016/j.esmoop.2020.04.023 32718488

[49] Bengtsson B, Greko C. Antibiotic resistance—consequences for animal health, welfare, and food production. Ups J Med Sci. 2014;119(2):96–102. 24678738 10.3109/03009734.2014.901445 PMC4034566

[50] Dyar OJ, et al. What is antimicrobial stewardship? Clin Microbiol Infect. 2017;23(11):793–8. 28882725 10.1016/j.cmi.2017.08.026

[51] Abbo LM, et al. Medical Students’ Perceptions and Knowledge About Antimicrobial Stewardship: How Are We Educating Our Future Prescribers? Clin Infect Dis. 2013;57(5):631–8. 23728148 10.1093/cid/cit370

[52] Luther VP, Ohl CA, Hicks LA. Antimicrobial Stewardship Education for Medical Students. Clin Infect Dis. 2013;57(9):1366–1366. 23893971 10.1093/cid/cit480

[53] MacDougall C et al. An Interprofessional Curriculum on Antimicrobial Stewardship Improves Knowledge and Attitudes Toward Appropriate Antimicrobial Use and Collaboration. Open Forum Infect Dis, 2017. 4(1). 10.1093/ofid/ofw225 PMC5414113 28480231

[54] Satterfield J, Miesner AR, Percival KM. The role of education in antimicrobial stewardship. J Hosp Infect. 2020;105(2):130–41. 32243953 10.1016/j.jhin.2020.03.028

[55] Efthymiou P, Gkentzi D, Dimitriou G. Knowledge, Attitudes and Perceptions of Medical Students on Antimicrobial Stewardship. Antibiot (Basel), 2020. 9(11). 10.3390/antibiotics9110821 PMC7698472 33213047

[56] Vickers H. International antibiotic resistance crisis. British Medical Journal Publishing Group; 2011.

[57] World Health Organisation. The evolving threat of antimicrobial resistance - Options for action. 2012 09/03/2024]; https://iris.who.int/bitstream/handle/10665/44812/?sequence=1

[58] Bal AM, Gould IM. Antibiotic stewardship: overcoming implementation barriers. Curr Opin Infect Dis. 2011;24(4):357–62. 21587070 10.1097/QCO.0b013e3283483262

[59] Johnson LS, MacDougall C, Trivedi KK. The Legislative Momentum of Antimicrobial Stewardship: the US Perspective. Curr Treat Options Infect Dis. 2016;8(2):93–101.

[60] Scheepers LN, Niesing CM, Bester P. Facilitators and barriers to implementing antimicrobial stewardship programs in public South African hospitals. Antimicrob Steward Healthc Epidemiol. 2023;3(1):e34. 36865702 10.1017/ash.2022.355 PMC9972532

[61] Australian Commission on Safety and Quality in Health Care. Antimicrobial stewardship education for clinicians. 2018 09/03/2024]; https://www.safetyandquality.gov.au/sites/default/files/migrated/Chapter5-Antimicrobial-stewardship-education-for-clinicians.pdf

[62] Alm RA, Lahiri SD. Narrow-Spectrum Antibacterial Agents-Benefits and Challenges. Antibiot (Basel), 2020. 9(7). 10.3390/antibiotics9070418 PMC7400354 32708925

[63] Karam G, et al. Antibiotic strategies in the era of multidrug resistance. Crit Care. 2016;20(1):136. 27329228 10.1186/s13054-016-1320-7 PMC4916531

[64] Melander RJ, Zurawski DV, Melander C. Narrow-Spectrum Antibact Agents Medchemcomm. 2018;9(1):12–21. 10.1039/c7md00528h PMC5839511 29527285

[65] Fishman N, America SfHEo, America IDSo. Policy statement on antimicrobial stewardship by the society for healthcare epidemiology of America (SHEA), the infectious diseases society of America (IDSA), and the pediatric infectious diseases society (PIDS). Infect Control Hosp Epidemiol. 2012;33(4):322–7. 22418625 10.1086/665010

[66] Castro-Sánchez E, et al. Mapping Antimicrobial Stewardship in Undergraduate Medical, Dental, Pharmacy, Nursing and Veterinary Education in the United Kingdom. PLoS ONE. 2016;11(2):e0150056. 26928009 10.1371/journal.pone.0150056 PMC4771156

[67] Chakravarty A et al. A Multicentric Survey of Indian Medical Students about their Knowledge and Perception on Antimicrobial Stewardship. J Pure Appl Microbiol, 2022. 16(2).

[68] Khan AKA, Banu G, K KR. Antibiotic Resistance and Usage-A Survey on the Knowledge, Attitude, Perceptions and Practices among the Medical Students of a Southern Indian Teaching Hospital. J Clin Diagn Res. 2013;7(8):1613–6. 24086854 10.7860/JCDR/2013/6290.3230 PMC3782911

[69] Melber DJ, Teherani A, Schwartz BS. A Comprehensive Survey of Preclinical Microbiology Curricula Among US Medical Schools. Clin Infect Dis. 2016;63(2):164–8. 27126343 10.1093/cid/ciw262

[70] Lim A, et al. Assessment of Antimicrobial Stewardship through objective structured clinical examination in pharmacy education. Int J Pharm Pract. 2023;31(6):646–9. 37410964 10.1093/ijpp/riad048

[71] Foral PA, et al. Education and Communication in an Interprofessional Antimicrobial Stewardship Program. J Am Osteopath Assoc. 2016;116(9):588–93. 27571295 10.7556/jaoa.2016.116

[72] Chetty S, et al. Interprofessional education in antimicrobial stewardship, a collaborative effort. JAC Antimicrob Resist. 2024;6(2):dlae054. 38562216 10.1093/jacamr/dlae054 PMC10984567

