# Supplementary material for: A global investigation into antimicrobial knowledge in medicine, pharmacy, nursing, dentistry and veterinary undergraduate students: A scoping review to inform future planetary health multidisciplinary education
Source: BMC Med Educ. 2024 Oct 29;24:1227. doi: 10.1186/s12909-024-06253-w (PMC11520657; doi:10.1186/s12909-024-06253-w)
Supplement: Supplementary file 1 — Supplementary Material 1 [file 12909_2024_6253_MOESM1_ESM.docx]

**Appendix I**

**Table 1: Articles used in the scoping review**

| **Article title** | **First author name** | **Country/geographical location** | **Year article was published** |
| --- | --- | --- | --- |
| Medical Students’ Perceptions and Knowledge About Antimicrobial Stewardship: How Are We Educating Our Future Prescribers? [1] | Lilian M. Abbo | USA | 2013 |
| Investigating Knowledge of Antibiotics, Antimicrobial Resistance and Antimicrobial Stewardship Concepts Among Final Year Undergraduate Pharmacy Students in Northern Nigeria [2] | Samirah N Abdu-Aguye | Nigeria | 2022 |
| Dental students' knowledge and attitudes towards antibiotic prescribing guidelines in Riyadh, Saudi Arabia [3] | Abdulrahman AboAlSamh | Saudi Arabia | 2018 |
| Knowledge and self-confidence of antibiotic resistance, appropriate antibiotic therapy, and antibiotic stewardship among pharmacy undergraduate students in three Asian countries [4] | Usman Abubakar | Indonesia, Malaysia, Pakistan | 2020 |
| Knowledge and self-reported confidence in antimicrobial stewardship programme among final year pharmacy undergraduate students in Malaysia and Nigeria [5] | Usman Abubakar | Malaysia, Nigeria | 2021 |
| Antibiotic Resistance and Usage—A Survey on the Knowledge, Attitude, Perceptions and Practices among the Medical Students of a Southern Indian Teaching Hospital [6] | Afzal Khan A K | India | 2013 |
| Knowledge, attitude and practice of B.Sc. Pharmacy students about antibiotics in Trinidad and Tobago [7] | Akram Ahmad | Trinidad, Tobago | 2015 |
| Comparison of knowledge and attitudes about antibiotics and resistance, and antibiotics self practicing between Bachelor of Pharmacy and Doctor of Pharmacy students in Southern India [8] | Akram Ahmad | India | 2015 |
| Knowledge about antibiotics and antibiotic resistance among health-related students in a Saudi University [9] | Zafar Akbar | Saudi Arabia | 2021 |
| Knowledge, attitudes, and practice with respect to antibiotic use among pharmacy students: a cross-sectional study [10] | W. AL-QEREM | Jordan | 2022 |
| Medical and dental students' knowledge and perceptions about antimicrobial stewardship: A call for educational enhancement [11] | Nada Alsaleh | Saudi Arabia | 2020 |
| Knowledge and attitudes regarding antibiotic use and resistance among nursing and dentistry students in Babylon University Iraq [12] | Sadiq Salam H. AL-Salih | Iraq | 2019 |
| Knowledge, Attitude, and Behavior about Antimicrobial Use and Resistance among Medical, Nursing and Pharmacy Students in Jordan: A Cross Sectional Study [13] | Ghaith M. Al-Taani | Jordan | 2022 |
| Knowledge, attitude and practice on antibiotic therapy among dental students-a pilot study [14] | Sudharsana Anandakumar | India | 2018 |
| Knowledge, attitudes, and practices of Egypt's future physicians towards antimicrobial resistance (KAP-AMR study): a multicenter cross-sectional study [15] | Ahmed Assar | Egypt | 2020 |
| Knowledge and perceptions about antibiotic resistance and prudent antibiotic prescribing among final year medical students in two African countries [16] | Bashar M Augie | South Africa, Nigeria | 2021 |
| Evaluation of Knowledge Regarding the Use of Antibiotics among Pharmacy Undergraduates in Japan [17] | Takuya Azechi | Japan | 2022 |
| Knowledge, attitudes and behaviours regarding antibiotics use among Cypriot university students: a multi-disciplinary survey [18] | Buket Baddal | Cyprus | 2022 |
| Awareness regarding antimicrobial resistance and confidence to prescribe antibiotics in dentistry: a cross-continental student survey [19] | Aya Bajalan | Norway, Canada, Brazil | 2022 |
| Nursing students' awareness and perceptions of nurses' role in antimicrobial stewardship [20] | St´ephane L. Bouchoucha | Australia | 2021 |
| Knowledge and perceptions of antimicrobial stewardship concepts among final year pharmacy students in pharmacy schools across South Africa [21] | Marisa Burger | South Africa | 2016 |
| A Multicentric Survey of Indian Medical Students about their Knowledge and Perception on Antimicrobial Stewardship [22] | Aparna Chakravarty | India | 2022 |
| Knowledge, attitudes and practices regarding antibiotic use and resistance among veterinary students in Bangladesh [23] | Lorraine Chapot | Bangladesh | 2022 |
| Lack of antibiotic knowledge and misuse of antibiotics by medical students in Mali: a cross-sectional study [24] | Jie Chen | Mali | 2020 |
| Perception, attitude, and knowledge regarding antimicrobial resistance, appropriate antimicrobial use, and infection control among future medical practitioners: A multicenter study [25] | Nuttagarn Chuenchom | Thailand | 2016 |
| Knowledge and practices of Indian dental students regarding the prescription of antibiotics and analgesics [26] | Astha Doshi | India | 2017 |
| A comparative knowledge, attitude, and practice study of antimicrobial use, self-medication and antimicrobial resistance among final year students of MBBS, BDS, and BSc nursing at a tertiary care hospital at Kannur [27] | Hemant Kumar Dutt | India | 2018 |
| Assessing the knowledge, attitudes and behaviors of human and animal health students towards antibiotic use and resistance: a pilot cross-sectional study in the UK [28] | Oliver James Dyar | United Kingdom | 2018 |
| Knowledge, attitudes, and beliefs of French medical students about antibiotic prescribing and resistance [29] | Oliver James Dyar | France | 2013 |
| Preparedness to prescribe antibiotics responsibly: a comparison between final year medical students in France and Sweden [30] | Oliver James Dyar | Sweden, France | 2019 |
| Do medical students feel prepared to prescribe antibiotics responsibly? Results from a cross-sectional survey in 29 European countries [31] | Oliver James Dyar | 29 European countries | 2018 |
| Managing responsible antimicrobial use: perspectives across the healthcare system [32] | Oliver James Dyar | Sweden | 2017 |
| European medical students and antibiotic stewardship: A multicentre survey of knowledge, attitudes and beliefs of antibiotic prescribing and antibiotic resistance [33] | Oliver James Dyar | 7 medical schools | 2013 |
| Knowledge, Attitudes, and Perceptions Associated With Antimicrobial Stewardship Among Veterinary Students: A Multi-Country Survey From Nigeria, South Africa, and Sudan [34] | Folorunso O. Fasina | Nigeria, South Africa, Sudan | 2020 |
| Pharmacy students' knowledge and attitudes about antibiotics in Kosovo [35] | Albina Fejza | Kosovo | 2016 |
| Knowledge, perception and preparedness of future prescribers about antimicrobial stewardship [36] | Jannatul Ferdoush | Bangladesh | 2016 |
| Assessing Knowledge, Beliefs, and Behaviors around Antibiotic Usage and Antibiotic Resistance among UK Veterinary Students: A Multi-Site, Cross-Sectional Survey [37] | Sarah E. Golding | United Kingdom | 2022 |
| Antibiotic literacy among Japanese medical students [38] | Hideharu Hagiya | Japan | 2020 |
| What do future pharmacists know about, and think of, antimicrobial stewardship? [39] | Lezley-Anne Hanna | United Kingdom | 2019 |
| Knowledge and Attitude of Antibiotic Prescription among Dental Students in Najaf City/Iraq [40] | Dunya Malhan Hanweet | Iraq | 2023 |
| A cross-sectional study evaluating the knowledge and beliefs about, and the use of antibiotics amongst Malaysian university students [41] | Mainul Haque | Malaysia | 2019 |
| Antibiotic prescribing and resistance: knowledge level of medical students of clinical years of University Sultan Zainal Abidin, Malaysia [42] | Mainul Haque | Malaysia | 2016 |
| Antimicrobial Prescribing Confidence and Knowledge Regarding Drug Resistance: Perception of Medical Students in Malaysia and the Implications [43] | Mainul Haque | Malaysia | 2022 |
| Antibiotic use: A cross-sectional study evaluating the understanding, usage and perspectives of medical students and pathfinders of a public defence university in Malaysia [44] | Mainul Haque | Malaysia | 2019 |
| Veterinary students’ knowledge and perceptions about antimicrobial stewardship and biosecurity—a national survey [45] | Laura Hardefeldt | Australia | 2018 |
| Understanding of Future Prescribers About Antimicrobial Resistance and Their Preparedness Towards Antimicrobial Stewardship Activities in Pakistan: Findings and Implications [46] | Khezar Hayat | Pakistan | 2022 |
| Understanding of pharmacy students towards antibiotic use, antibiotic resistance and antibiotic stewardship programs: A cross-sectional study from Punjab, Pakistan [47] | Khezar Hayat | Pakistan | 2021 |
| Knowledge regarding antibiotic use among students of three medical schools in Medellin, Colombia: a cross-sectional study [48] | Luis Felipe Higuita-Gutiérrez | Colombia | 2020 |
| Knowledge, attitude, and practice regarding antibiotic use and resistance among medical students in Colombia: a cross-sectional descriptive study [49] | Luis Felipe Higuita-Gutiérrez | Colombia | 2020 |
| Antibiotics and antimicrobial resistance: Evaluation of the knowledge, attitude, and perception among students and faculty within US dental schools [50] | Magdalena Holz | USA | 2020 |
| Insight of medical students of clinical years to antimicrobials prescribing and resistance in private medical school, Chittagong, Bangladesh [51] | Rozina Hoque | Bangladesh | 2016 |
| Survey of the Knowledge, Attitudes and Practice towards Antibiotic Use among Prospective Antibiotic Prescribers in Serbia [52] | Olga Horvat | Serbia | 2022 |
| Serbian students' knowledge, attitudes and behaviour towards antibiotic use: is there room for improvement? [53] | Olga Horvat | Serbia | 2020 |
| Knowledge, attitude and practice of antibiotics: a questionnaire study among 2500 Chinese students [54] | Ying Huang | China | 2013 |
| Assessing knowledge and perception regarding antimicrobial stewardship and antimicrobial resistance in university students of Pakistan: Findings and implications [55] | Iltaf Hussain | Pakistan | 2021 |
| Impact of Antibiotic Stewardship Learning Session on Knowledge and Attitude of Undergraduate Medical Students [56] | Mazhar Hussain | Pakistan | 2021 |
| Knowledge, Attitude, and Practice with Respect to Antibiotic Use among Chinese Medical Students: A Multicentre Cross-Sectional Study [57] | Yanhong Hu | China | 2018 |
| A survey on knowledge, attitude, and perception of antibiotic resistance and usage among dental students [58] | Mokshi R. Jain | India | 2019 |
| Knowledge, attitude and practices on antimicrobial resistance and usage among medical students in a Malaysian medical university [59] | Heethal Jaiprakash | Malaysia | 2020 |
| Knowledge, attitude and practice of antibiotic use among university students: a cross sectional study in UAE [60] | Ammar Jairoun | UAE | 2019 |
| Understanding of antibiotic use and resistance among final-year pharmacy and medical students: A pilot study [61] | Shazia Qasim Jamshed | Malaysia | 2013 |
| Change in the Perception of Oral Antibiotics Among Medical Students After Participating in a Parenteral-to-Oral Conversion Program for Highly Bioavailable Antibiotics [62] | Wooyoung Jang | South Korea | 2022 |
| Perceptions and preparedness of senior medical students about antimicrobial stewardship programs: are we providing adequate training to future prescribers? [63] | Wenjing Ji | China | 2023 |
| Knowledge and attitudes of doctor of pharmacy students regarding the appropriate use of antimicrobials [64] | Julie Ann Justo | USA | 2014 |
| Knowledge, attitude and perception on antimicrobial use and antimicrobial resistance among final year medical students in the College of Medicine, Malawi [65] | Alfred Kamoto | Malawi | 2020 |
| Knowledge, attitude, perception and practice of antibiotics usage among the pharmacy students [66] | Geetha Kandasamy | Saudi Arabia | 2020 |
| Antimicrobial resistance and rational use of medicine: knowledge, perceptions, and training of clinical health professions students in Uganda [67] | Andrew Marvin Kanyike | Uganda | 2022 |
| Exploring Undergraduate Pharmacy Students Perspectives Towards Antibiotics Use, Antibiotic Resistance, and Antibiotic Stewardship Programs Along With the Pharmacy Teachers’ Perspectives: A Mixed-Methods Study From Pakistan [68] | Faiz Ullah Khan | Pakistan | 2021 |
| Knowledge about antibiotic resistance among dental students in Chengalpattu district, Tamil Nadu - A cross-sectional study [69] | K Indrapriyadharshini | India | 2021 |
| Mapping knowledge and comprehension of antimicrobial stewardship and biosecurity among veterinary students [70] | Zorana Kovacevic | Serbia, Croatia | 2020 |
| Preparedness of medicine and pharmacy students in Sub-Saharan Africa to prescribe antibiotics appropriately in an era of antimicrobial resistance [71] | Margaret Lubwama | Kenya | 2020 |
| Knowledge, attitudes, and perceptions about antibiotic use and antimicrobial resistance among final year undergraduate medical and pharmacy students at three universities in East Africa [72] | Margaret Lubwama | East Africa | 2021 |
| Knowledge, perception, and educational status of antimicrobial resistance among Chinese medical students [73] | Zhao, A | China | 2019 |
| Assessing knowledge, perception and attitudes about antibiotics among final year pharmacy undergraduates in Sri Lanka [74] | Zawahir, S | Sri Lanka | 2017 |
| Impact of Infectious Diseases training in the perception of antibiotic resistance and rational use of antibiotics among Spanish medical students – a cross-sectional study [75] | Yuste, J. R | Spain | 2022 |
| Pharmacy students knowledge and attitudes towards antibiotic use: A cross-sectional study [76] | Yilmaz, Z | Turkey | 2023 |
| Attitudes and perceptions regarding antimicrobial use and resistance among medical students in Central China [77] | Yang, K | China | 2016 |
| A Comparative Study about Knowledge, Attitude, Practice of Antibiotic Use and Perceptions of the Possible Causes of Resistance between Final Year Undergraduate Students and Postgraduate Pharmacy Students [78] | Yahya, R. N | Iraq | 2021 |
| Survey of antibiotic knowledge amongst final year medical students [79] | Wright, E P | United Kingdom | 2004 |
| Dental students compliance with antibiotic prescribing guidelines for dental infections in children [80] | Wong, Y C | Malaysia, Hong Kong, Taiwan | 2016 |
| Appropriate antibiotic use and antimicrobial resistance: knowledge, attitudes and behaviour of medical students and their needs and preferences for learning [81] | Wiese-Posselt, M | Germany | 2023 |
| Antimicrobial knowledge and confidence amongst final year medical students in Australia [82] | Weier, N | Australia | 2017 |
| South African medical students' perceptions and knowledge about antibiotic resistance and appropriate prescribing: Are we providing adequate training to future prescribers? [83] | Waserman, S | South Africa | 2017 |
| Knowledge, attitude and practice of antibiotics among medical students in Vietnam [84] | Vo, T Q | Vietnam | 2018 |
| Knowledge, Perceptions, and Perspectives of Medical Students Regarding the Use of Antibiotics and Antibiotic Resistance: A Qualitative Research in Galicia, Spain [85] | Vazquez-Lago, J M | Spain | 2023 |
| Antibiotic prescribing in DR Congo: a knowledge, attitude and practice survey among medical doctors and students [86] | Thriemer, K | DR Congo | 2013 |
| Knowledge and perceptions of medical students regarding antibiotic use [87] | Tayyab, K | Pakistan | 2017 |
| Awareness of antibiotic use and antibiotic resistance amongst dental students [88] | Tariq, K | Pakistan | 2021 |
| Knowledge of antibiotics and antimicrobial resistance amongst final year dental students of Polish medical schools-A cross-sectional study [89] | Struzycka, I | Poland | 2019 |
| Knowledge-based attitudes of medical students in antibiotic therapy and antibiotic resistance. A cross-sectional study [90] | Sobierajski, T | Poland | 2021 |
| Antimicrobial and Antibiotic Resistance from the Perspective of Polish Veterinary Students: An Inter-University Study [91] | Sobierajski, T | Poland | 2022 |
| Implication of the knowledge and perceptions of veterinary students of antimicrobial resistance for future prescription of antimicrobials in animal health, South Africa [92] | Smith, P W | South Africa | 2019 |
| Knowledge, attitude and practice on antibiotics use and its resistance among medical students in a tertiary care hospital [93] | Shrestha, R | Nepal | 2019 |
| A cross sectional survey of knowledge, attitude and practice of antibiotic use among dental and paramedical students [94] | Sharon Keziah, V | India | 2020 |
| Prevalence of self-medication of antibiotics among 2nd-year medical students and their knowledge about antibiotic resistance [95] | Sharma, N | India | 2020 |
| Knowledge, attitude and perception of medical and dental undergraduates about antimicrobial stewardship [96] | Sharma, K | India | 2015 |
| Knowledge, Attitude and Practice Towards Antibiotic Use and Antibiotic Resistance among Medical Students: A Cross-Sectional Study [97] | Sharma, A | India | 2022 |
| Antibiotic use: A cross-sectional survey assessing the knowledge, attitudes, and practices amongst students of Punjab, Pakistan [98] | Shah, S | Pakistan | 2022 |
| Knowledge, Attitude, and Practice Associated with Antibiotic Use among University Students: A Survey in Nepal [99] | Shah, P | Nepal | 2019 |
| Knowledge and attitude towards antimicrobial resistance among final year undergraduate paramedical students at University of Gondar, Ethiopia [100] | Seid, M A | Ethiopia | 2018 |
| Evaluation of Healthcare Students' Knowledge on Antibiotic Use, Antimicrobial Resistance and Antimicrobial Stewardship Programs and Associated Factors in a Tertiary University in Ghana: Findings and Implications [101] | Sefah, I A | Ghana | 2022 |
| How decisions are made: Antibiotic stewardship in dentistry [102] | Schneider-Smith, E G | USA | 2023 |
| Antibiotic Use: A cross-sectional survey assessing the knowledge, attitudes and practices amongst students of a school of medicine in Italy [103] | Scaioli, G | Italy | 2015 |
| Survey on knowledge towards antibiotics among the nursing students [104] | SatishKumar, BP | India | 2011 |
| Evaluation of the education in antimicrobial stewardship and resistance by students of two Belgian Faculties of Veterinary Medicine [105] | Sarino, N | Belgium | 2022 |
| A cross-sectional study to evaluate the knowledge and attitude of medical students concerning antibiotic usage and antimicrobial resistance [106] | Sannathimmappa , M B | India | 2021 |
| Perspective of Spanish medical students regarding undergraduate education in infectious diseases, bacterial resistance and antibiotic use [107] | Sanchez-Fabra, D | Spain | 2019 |
| Medical and pharmacy students' knowledge, attitude and perception concerning antimicrobial use and resistance in Pakistan [108] | Saleem, Z | Pakistan | 2019 |
| Investigating knowledge regarding antibiotics among pharmacy and allied health sciences students in a Sri Lankan university [109] | Sakeena, M H F | Sri Lanka | 2018 |
| Investigating knowledge regarding antibiotics and antimicrobial resistance among pharmacy students in Australian universities [110] | Sakeena, M H F | Australia | 2021 |
| A comparative study regarding antibiotic consumption and knowledge of antimicrobial resistance among pharmacy students in Australia and Sri Lanka [111] | Sakeena, M H F | Australia & Sri Lanka | 2019 |
| Attitudes and knowledge regarding antimicrobial use and resistance among pharmacy and medical students at the University of Split, Croatia [112] | Rusic, D | Croatia | 2018 |
| Confidence in antibiotic prescribing intentions among senior medical students in India [113] | Ritchie, O | India | 2020 |
| Awareness of Antibiotic Resistance among Medical Students in Kerala State, India: A Cross-Sectional Study [114] | Reena, A P | India | 2022 |
| Perception, attitude, knowledge and learning style preference on challenges of antimicrobial resistance and antimicrobial overuse among first year doctors in training and final year medical students [115] | Rattanaumpawan, P | Thailand | 2019 |
| Impact of Hospital Acquired Infection and Antibiotic Resistance Awareness Campaign on Knowledge Attitude and Practices of Medical Undergraduates in a Tertiary Care Teaching Hospital, India [116] | Rajni, E | India | 2020 |
| Evaluation of the understanding of antibiotic resistance among Malaysian pharmacy students at public universities: An exploratory study [117] | Rajiah, K | Malaysia | 2015 |
| Understanding of Final Year Medical, Pharmacy and Nursing Students in Pakistan towards Antibiotic Use, Antimicrobial Resistance and Stewardship: Findings and Implications [118] | Raees, I | Pakistan | 2023 |
| Nursing students' knowledge and awareness of antibiotic use, resistance and stewardship: A descriptive cross-sectional study [119] | Rabano-Blanco, A | Spain | 2019 |
| Exploring medical and veterinary student perceptions and communication preferences related to antimicrobial resistance in Ontario, Canada using qualitative methods [120] | Primeau, C A | Canada | 2023 |
| Survey on knowledge, attitude and practice of antimicrobial resistance among dental students [121] | PreethiMariona, R | India | 2017 |
| The knowledge and perception of pharmacy students and recent graduates in IPSF toward antimicrobial stewardship [122] | Popoola, O , O | Africa, Europe, Asia, East Mediterranean, America | 2022 |
| Survey of Practices, Knowledge and Attitude Concerning Antibiotics and Antimicrobial Resistance among Medical University Students [123] | Phagava, H | Georgia | 2019 |
| Emerging causes for antibiotic resistance: second-year medical undergraduate's perspective: A cross-sectional study [124] | Padmanabha, T S | India | 2018 |
| Self-Medication and Antimicrobial Resistance: A Survey of Students Studying Healthcare Programmes at a Tertiary Institution in Ghana [125] | Owusu-Ofori, A K | Ghana | 2021 |
| Antibiotic prescribing and resistance: knowledge among Latin American medical students during Antibiotic Awareness Week 2016 [126] | Ortiz-Martinez, Y | Columbia (& other latin American countries) | 2017 |
| Antimicrobial stewardship: Perception and familiarity of future prescribers in a tertiary health institution in Northern Nigeria [127] | Olorukooba, A A | Nigeria | 2020 |
| Knowledge of antibiotic use and resistance among students of a medical school in Nigeria [128] | Okedo-Alex, I | Nigeria | 2019 |
| Knowledge of antimicrobial resistance among veterinary students and their personal antibiotic use practices: A national cross-sectional survey [129] | Odetokun, I A | Nigeria | 2019 |
| Assessment of the knowledge, attitudes and perceived quality of education about antimicrobial use and resistance of medical students in Zambia, Southern Africa [130] | Nowbuth, A A | Zambia | 2023 |
| Faces of resistance: Using real-world patients and their advocates to teach medical students about antimicrobial stewardship [131] | Nori | USA | 2019 |
| Knowledge and attitudes towards antibiotic use and resistance among undergraduate healthcare students at University of Rwanda [132] | Nisabwe, L | Rwanda | 2020 |
| How are we educating future physicians and pharmacists in pakistan? A survey of the medical and pharmacy student's perception on learning and preparedness to assume future roles in antibiotic use and resistance [133] | Mubarak, N | Pakistan | 2021 |
| A survey of knowledge, attitudes, and beliefs of medical students concerning antimicrobial use and resistance [134] | Minen, M T | USA | 2010 |
| Knowledge, attitude, and practice associated with antimicrobial resistance among medical students between 2017 and 2022: A survey in East China [135] | Min, S | China | 2022 |
| Assessment of antibiotic knowledge among final year pharmacy students at Baghdad university [136] | Mikhael, E M | Iraq | 2019 |
| Factors most influencing antibiotic stewardship program and comparison of prefinal- and final-year undergraduate medical students [137] | Meher, B R | India | 2020 |
| Knowledge, attitude and practice towards antibiotic use: An interventional study among medical and dental undergraduates [138] | Mate, V H | India | 2022 |
| Survey of the knowledge and use of antibiotics among medical and veterinary health professionals and students in Portugal [139] | Marta-Costa, A | Portugal | 2021 |
| Assessment of knowledge, attitudes, and practices about antibiotic resistance among medical students in Central India [140] | Marskole, P | India | 2022 |
| Antibiotic Use and Resistance: A Cross-Sectional Study Exploring Knowledge, Attitudes and Practices among Medical, Dental, Pharmacy and Nursing Students [141] | Manikanta, K N | India | 2022 |
| KAP on Antibiotic Usage and Resistance among Second Professional Medical Students [142] | Mangal, N | India | 2023 |
| Enhancing Medical Students' Confidence and Knowledge in Antibiotic Prescription and Administration through Virtual Education: A Quasi-Experimental Study [143] | Malli, I A | Saudi Arabia | 2023 |
| An interprofessional curriculum on antimicrobial stewardship improves knowledge and attitudes toward appropriate antimicrobial use and collaboration [144] | MacDougall, C | USA | 2017 |

**Table 2: Year of publication**

| **Year Published** | **Frequency** |
| --- | --- |
| 2004 | 1 |
| 2010 | 1 |
| 2011 | 1 |
| 2013 | 7 |
| 2014 | 1 |
| 2015 | 5 |
| 2016 | 8 |
| 2017 | 8 |
| 2018 | 13 |
| 2019 | 22 |
| 2020 | 22 |
| 2021 | 18 |
| 2022 | 26 |
| 2023 | 11 |
| Total | 144 articles |

**Table 3: Geographical location of publication**

| **Geographical location** | **Frequency** |
| --- | --- |
| 29 European countries | 1 |
| 7 medical schools | 1 |
| Africa, Europe, Asia, East Mediterranean, America | 1 |
| Australia | 4 |
| Australia & Sri Lanka | 1 |
| Bangladesh | 3 |
| Belgium | 1 |
| Canada | 1 |
| China | 6 |
| Colombia | 2 |
| Columbia (& other Latin American countries) | 1 |
| Croatia | 1 |
| Cyprus | 1 |
| DR Congo | 1 |
| East Africa | 1 |
| Egypt | 1 |
| Ethiopia | 1 |
| France | 1 |
| Georgia | 1 |
| Germany | 1 |
| Ghana | 2 |
| India | 24 |
| Indonesia, Malaysia, Pakistan | 1 |
| Iraq | 4 |
| Italy | 1 |
| Japan | 2 |
| Jordan | 2 |
| Kenya | 1 |
| Kosovo | 1 |
| Malawi | 1 |
| Malaysia | 7 |
| Malaysia, Hong Kong, Taiwan | 1 |
| Malaysia, Nigeria | 1 |
| Mali | 1 |
| Nepal | 2 |
| Nigeria | 4 |
| Nigeria, South Africa, Sudan | 1 |
| Norway, Canada, Brazil | 1 |
| Pakistan | 11 |
| Poland | 3 |
| Portugal | 1 |
| Rwanda | 1 |
| Saudi Arabia | 5 |
| Serbia | 2 |
| Serbia, Croatia | 1 |
| South Africa | 3 |
| South Africa, Nigeria | 1 |
| South Korea | 1 |
| Spain | 4 |
| Sri Lanka | 3 |
| Sweden, France | 1 |
| Thailand | 2 |
| Trinidad, Tobago | 1 |
| Turkey | 1 |
| UAE | 1 |
| Uganda | 1 |
| United Kingdom | 4 |
| USA | 7 |
| Vietnam | 1 |
| Zambia | 1 |
| Total | 144 articles |

**Table 4: Study type and design**

| **Study Type and Design** | **Frequency** |
| --- | --- |
| Quantitative: Cross-sectional study | 138 |
| Qualitative: Focus groups | 2 |
| Mixed:  Quantitative: cross-sectional study Qualitative: semi-structured interviews and/or focus groups | 4 |
| Total | 144 articles |

**Table 5: Discipline count**

| **Number of Disciplines** | **Frequency** | **%** |
| --- | --- | --- |
| 1 discipline | 115 | 80% |
| 2 disciplines | 17 | 12% |
| 3 disciplines | 10 | 7% |
| 4 disciplines | 1 | 1% |
| 5 disciplines | 1 | 1% |
| Total | 144 | 100% |

**Table 6: Discipline representation in the articles**

| **Discipline** | **Frequency** |
| --- | --- |
| Medicine | 89 |
| Pharmacy | 42 |
| Nursing | 16 |
| Dentistry | 26 |
| Veterinary | 15 |

**Table 7: Familiarity with AMS**

| **% of students familiar with AMS** | **Frequency of studies** |
| --- | --- |
| 0 - 10 | 6 |
| 10 - 20 | 7 |
| 20 - 30 | 3 |
| 30 - 40 | 3 |
| 40 - 50 | 6 |
| 50 - 60 | 2 |
| 60 - 70 | 6 |
| 70 - 80 | 0 |
| 80 - 90 | 2 |
| 90 - 100 | 3 |
| Total | 38 |

**Table 8: Familiarity with AMR**

| **% of students familiar with AMR** | **Frequency of studies** |
| --- | --- |
| 0 - 10 | 1 |
| 10 - 20 | 2 |
| 20 - 30 | 0 |
| 30 - 40 | 1 |
| 40 - 50 | 5 |
| 50 - 60 | 11 |
| 60 - 70 | 4 |
| 70 - 80 | 7 |
| 80 - 90 | 25 |
| 90 - 100 | 35 |
| Total | 91 |

**Table 9: Disciplines’ familiarity with AMR and AMS**

| **Medicine** | | **Pharmacy** | | **Nursing** | | **Dentistry** | | **Veterinary** | |
| --- | --- | --- | --- | --- | --- | --- | --- | --- | --- |
| AMS % (n=18) | AMR % (n=41) | AMS % (n=8) | AMR % (n=16) | AMS % (n=2) | AMR % (n=2) | AMS % (n=0) | AMR % (n=9) | AMS % (n=4) | AMR % (n=8) |
| 35.5% | 79.4% | 50.2% | 79.2% | 69.5% | 78.8% | N/A | 76.2% | 45.9% | 73.7% |

**Table 10: Disciplines’ understanding of factors contributing to AMR**

| **Medicine** | | **Pharmacy** | | **Nursing** | | **Dentistry** | | **Veterinary** | |
| --- | --- | --- | --- | --- | --- | --- | --- | --- | --- |
| Inappropriate antimicrobial use % (n=36) | Inappropriate broad-spectrum antimicrobial use % (n=21) | Inappropriate antimicrobial use % (n=6) | Inappropriate broad-spectrum antimicrobial use % (n=8) | Inappropriate antimicrobial use % (n=3) | Inappropriate broad-spectrum antimicrobial use % (n=1) | Inappropriate antimicrobial use % (n=6) | Inappropriate broad-spectrum antimicrobial use % (n=1) | Inappropriate antimicrobial use % (n=5) | Inappropriate broad-spectrum antimicrobial use % (n=1) |
| 82.0% | 67.4% | 80.4% | 73.8% | 75.0% | 51.3% | 81.6% | 72.0% | 86.9% | 87.3% |

**Table 11: Disciplines’ knowledge of antimicrobial prescribing**

| **Medicine** | | | | **Pharmacy** | | | | **Nursing** | | | |
| --- | --- | --- | --- | --- | --- | --- | --- | --- | --- | --- | --- |
| Knowing when to start antimicrobials % (n=11) | Selecting the most appropriate antimicrobial % (n=16) | Choosing appropriate dosage regimen % (n=15) | Intravenous to oral switch % (n=21) | Knowing when to start antimicrobials % (n=2) | Selecting the most appropriate antimicrobial % (n=2) | Choosing appropriate dosage regimen % (n=3) | Intravenous to oral switch % (n=2) | Knowing when to start antimicrobials % (n=0) | Selecting the most appropriate antimicrobial % (n=1) | Choosing appropriate dosage regimen % (n=1) | Intravenous to oral switch % (n=0) |
| 85.7% | 68.6% | 48.6% | 82.5% | 74.4% | 67.4% | 62.8% | 58.8% | N/A | 2.9% | 14.6% | N/A |

| **Dentistry** | | | | **Veterinary** | | | |
| --- | --- | --- | --- | --- | --- | --- | --- |
| Knowing when to start antimicrobials % (n=0) | Selecting the most appropriate antimicrobial % (n=1) | Choosing appropriate dosage regimen % (n=2) | Intravenous to oral switch % (n=0) | Knowing when to start antimicrobials % (n=0) | Selecting the most appropriate antimicrobial % (n=2) | Choosing appropriate dosage regimen % (n=0) | Intravenous to oral switch % (n=0) |
| N/A | 71.1% | 55.0% | N/A | N/A | 37.9% | N/A | N/A |

**Table 12: Disciplines’ appreciation of the importance of appropriate antimicrobial prescribing, AMR and AMS**

| **Medicine** | | | | **Pharmacy** | | | | **Nursing** | | | |
| --- | --- | --- | --- | --- | --- | --- | --- | --- | --- | --- | --- |
| Knowledge of antimicrobials, AMR and AMS is important in the career % (n=11) | Need for further education on antimicrobial prescribing % (n=28) | Need for further education on AMR % (n=11) | Need for further education on AMS % (n=1) | Knowledge of antimicrobials, AMR and AMS is important in the career % (n=3) | Need for further education on antimicrobial prescribing % (n=6) | Need for further education on AMR % (n=4) | Need for further education on AMS % (n=4) | Knowledge of antimicrobials, AMR and AMS is important in the career % (n=1) | Need for further education on antimicrobial prescribing % (n=1) | Need for further education on AMR % (n=1) | Need for further education on AMS % (n=1) |
| 86.4% | 83.0% | 83.0% | 69.0% | 95.4% | 92.7% | 91.0% | 90.7% | 97.1% | 97.4% | 97.4% | 97.4% |

| **Dentistry** | | | | **Veterinary** | | | |
| --- | --- | --- | --- | --- | --- | --- | --- |
| Knowledge of antimicrobials, AMR and AMS is important in the career % (n=0) | Need for further education on antimicrobial prescribing % (n=3) | Need for further education on AMR % (n=2) | Need for further education on AMS % (n=0) | Knowledge of antimicrobials, AMR and AMS is important in the career % (n=0) | Need for further education on antimicrobial prescribing % (n=3) | Need for further education on AMR % (n=1) | Need for further education on AMS % (n=0) |
| N/A | 81.3% | 84.8% | N/A | N/A | 66.8% | 87.0% | N/A |

**Table 13: Studies that reported on students’ understanding of when antimicrobials are not indicated**

| **Article title** | **First author name** | **Country/**  **geographical location** | **Year article was published** | **Key findings** |
| --- | --- | --- | --- | --- |
| Medical Students’ Perceptions and Knowledge About Antimicrobial Stewardship: How Are We Educating Our Future Prescribers? | Lilian M. Abbo | USA | 2013 | “59.0% of the students were able to identify scenarios with potential for unnecessary use of antimicrobials.” |
| Knowledge and self-confidence of antibiotic resistance, appropriate antibiotic therapy, and antibiotic stewardship among pharmacy undergraduate students in three Asian countries | Usman Abubakar | Indonesia, Malaysia, Pakistan | 2020 | “66.4% of students were able to identify infections that do not require antibiotic therapy.” |
| Antibiotic Resistance and Usage—A Survey on the Knowledge, Attitude, Perceptions and Practices among the Medical Students of a Southern Indian Teaching Hospital | Afzal Khan A K | India | 2013 | “>85% of the respondents agreed that an indiscriminate and an injudicious use of antibiotics could lead to an ineffective treatment, increased adverse effects, the emergence of bacterial resistance and an additional burden of medical costs to the patient.”  “77.3% respondents were aware that bacteria were not responsible for causing colds and flu.” |
| Knowledge, attitude and practice of B.Sc. Pharmacy students about antibiotics in Trinidad and Tobago | Akram Ahmad | Trinidad, Tobago | 2015 | “62.03% correctly answered bacteria are not responsible for common cold and flu.” |
| Knowledge, Attitude, and Behavior about Antimicrobial Use and Resistance among Medical, Nursing and Pharmacy Students in Jordan: A Cross Sectional Study | Ghaith M. Al-Taani | Jordan | 2022 | “85.5% realised that antibiotics are not effective against viruses, and 76.7% realised that they are not effective against cold and flu.” |
| A Multicentric Survey of Indian Medical Students about their Knowledge and Perception on Antimicrobial Stewardship | Aparna Chakravarty | India | 2022 | “90.8% students responded correctly that the most common cause of fever and upper respiratory symptoms in children were due to viral illness and did not require antibiotics.” |
| Assessing the knowledge, attitudes and behaviors of human and animal health students towards antibiotic use and resistance: a pilot cross-sectional study in the UK | Oliver James Dyar | United Kingdom | 2018 | “Most students knew that antibiotics kill both commensal and pathogenic bacteria (88%), and that overuse of antibiotics makes them less effective (96%). Very few (1%) thought that antibiotics killed viruses. Most students (92%) agreed that most coughs, colds and sore throats get better on their own without the need for antibiotics, but 25% of dentistry students still thought that antibiotics were effective against colds. All students were aware that bacteria can become resistant to antibiotics, but many also believed that humans or animals can become resistant.” |
| Knowledge, attitude, and practice regarding antibiotic use and resistance among medical students in Colombia: a cross-sectional descriptive study | Luis Felipe Higuita-Gutiérrez | Colombia | 2020 | “Regarding practice, 11.8% (n = 63) of the students stated that antibiotics are effective for treating viral infections, 11.6% (n = 62) stated that antibiotics are used to treat flu or the common cold, 12.2% (n = 65) stated that antibiotics should be discontinued when symptoms disappear, 8% (n = 43) stated that antibiotics are the first-choice treatment in the presence of cough and sore throat, and 28.5% (n = 152) recognized that antibiotic resistance is a multifactorial problem but do not act on it because individual actions would have little impact.” |
| Survey of the Knowledge, Attitudes and Practice towards Antibiotic Use among Prospective Antibiotic Prescribers in Serbia | Olga Horvat | Serbia | 2022 | “Although 81.3% of the total sample stated antibiotics cannot be used for the treatment of common cold, it is surprising that this percentage was significantly lower among students of veterinary medicine (63%) compared to students of medicine (85.1%) and dentistry (91%).” |
| Assessing knowledge and perception regarding antimicrobial stewardship and antimicrobial resistance in university students of Pakistan: Findings and implications | Iltaf Hussain | Pakistan | 2021 | “More than half of the participants correctly identified that antibiotics cannot kill viruses (59.4%). The veterinary students showed good knowledge regarding the contribution of poorly designed dosing regimens to AMR and as well as the contribute of the use of antibiotics for viruses/viral diseases compared to the students of pharmaceutical and biological sciences.” |
| Knowledge, Attitude, and Practice with Respect to Antibiotic Use among Chinese Medical Students: A Multicentre Cross-Sectional Study | Yanhong Hu | China | 2018 | “More than 60% of the medical students answered correctly that the common cold was a self-limiting disease not requiring antibiotics.”   “47% of medical students did not agree that antibiotics could reduce the symptoms of the common cold.” |
| Understanding of antibiotic use and resistance among final-year pharmacy and medical students: A pilot study | Shazia Qasim Jamshed | Malaysia | 2013 | “Regarding understanding of antibiotic use, the majority of the students answered most of the questions correctly, e.g. antibiotics cannot treat influenza or common cold and cough, antibiotics might develop allergy in susceptible individuals, etc.” |
| Knowledge of antibiotics and antimicrobial resistance amongst final year dental students of Polish medical schools-A cross-sectional study | Struzycka, I | Poland | 2019 | “The vast majority of respondents knew that antibiotics are ineffective in treating viral diseases, such as the flu and the common cold (91% and 87%, respectively). About 54% expressed the opinion that dentists overprescribe antibiotics. One‐tenth thought that they can be used for the treatment of flu (7%) and common cold (11%).” |
| Antimicrobial and Antibiotic Resistance from the Perspective of Polish Veterinary Students: An Inter-University Study | Sobierajski, T | Poland | 2022 | “Almost all respondents knew that antibiotics are ineffective for treating infections caused by viruses (n = 451, 96.6%) and are effective against bacteria (n = 438, 93.8%).” |
| Antibiotic Use: A cross-sectional survey assessing the knowledge, attitudes and practices amongst students of a school of medicine in Italy | Scaioli, G | Italy | 2015 | “Around 20% of the sample stated that antibiotics are appropriate for viral infections.”   “Regarding the knowledge about antibiotic use, almost all the participants were aware that antibiotics are useful for treating bacterial infections (95.2%) and that these drugs are not indicated for every kind of pain and inflammation (96.6%). Moreover, a relatively low percentage of the sample (83.2%) was conscious that antimicrobial drugs are not appropriate for viral infections.” |
| A cross-sectional study to evaluate the knowledge and attitude of medical students concerning antibiotic usage and antimicrobial resistance | Sannathimmappa, M B | India | 2021 | “Approximately 25% had a misconception that antibiotics can be used to treat either viral infections.” |
| Impact of Hospital Acquired Infection and Antibiotic Resistance Awareness Campaign on Knowledge Attitude and Practices of Medical Undergraduates in a Tertiary Care Teaching Hospital, India | Rajni, E | India | 2020 | “61.6% respondents in the present study believed that antibiotics do not speed up the recovery of common cold. This indirectly showcased their knowledge on the fact that diseases like influenza and common cold are not of bacterial origin and hence do not need antimicrobial drugs.” |
| Understanding of Final Year Medical, Pharmacy and Nursing Students in Pakistan towards Antibiotic Use, Antimicrobial Resistance and Stewardship: Findings and Implications | Raees, I | Pakistan | 2023 | “81.8% (n=1023) knew that antibiotics are not useful in treating viral infections.” |
| Survey of Practices, Knowledge and Attitude Concerning Antibiotics and Antimicrobial Resistance among Medical University Students | Phagava, H | Georgia | 2019 | “33.02% believe antibiotics can cure viral infections.” |
| Self-Medication and Antimicrobial Resistance: A Survey of Students Studying Healthcare Programmes at a Tertiary Institution in Ghana | Owusu-Ofori, A K | Ghana | 2021 | “92.0% (n = 232) and 62.7% (n = 158) of the participants who knew about antibiotics (n = 252) correctly answered that antibiotics can be used to cure bacterial infections and have no effect on viral infections, respectively.” |

**References**

1. Abbo, L.M., et al., *Medical Students’ Perceptions and Knowledge About Antimicrobial Stewardship: How Are We Educating Our Future Prescribers?* Clinical Infectious Diseases, 2013. **57**(5): p. 631-638.

2. Abdu-Aguye, S.N., et al., *Investigating knowledge of antibiotics, antimicrobial resistance and antimicrobial stewardship concepts among final year undergraduate pharmacy students in northern Nigeria.* Integrated Pharmacy Research and Practice, 2022: p. 187-195.

3. AboAlSamh, A., et al., *Dental Students' Knowledge and Attitudes towards Antibiotic Prescribing Guidelines in Riyadh, Saudi Arabia.* Pharmacy (Basel), 2018. **6**(2).

4. Abubakar, U., et al., *Knowledge and self-confidence of antibiotic resistance, appropriate antibiotic therapy, and antibiotic stewardship among pharmacy undergraduate students in three Asian countries.* Currents in Pharmacy Teaching and Learning, 2020. **12**(3): p. 265-273.

5. Abubakar, U., et al., *Knowledge and self-reported confidence in antimicrobial stewardship programme among final year pharmacy undergraduate students in Malaysia and Nigeria.* Pharmacy Education, 2021. **21**: p. 298-305.

6. AK, A.K., G. Banu, and K. Reshma, *Antibiotic resistance and usage—a survey on the knowledge, attitude, perceptions and practices among the medical students of a Southern Indian teaching hospital.* Journal of clinical and diagnostic research: JCDR, 2013. **7**(8): p. 1613.

7. Ahmad, A., et al., *Knowledge, attitude and practice of B. Sc. Pharmacy students about antibiotics in Trinidad and Tobago.* Journal of research in pharmacy practice, 2015. **4**(1): p. 37-41.

8. Ahmad, A., et al., *Comparison of knowledge and attitudes about antibiotics and resistance, and antibiotics self-practicing between Bachelor of Pharmacy and Doctor of Pharmacy students in Southern India.* Pharmacy practice, 2015. **13**(1).

9. Akbar, Z., et al., *Knowledge about antibiotics and antibiotic resistance among health-related students in a Saudi University.* The Journal of Infection in Developing Countries, 2021. **15**(07): p. 925-933.

10. Al-Qerem, W., et al., *Knowledge, attitudes, and practice with respect to antibiotic use among pharmacy students: a cross-sectional study.* European Review for Medical & Pharmacological Sciences, 2022. **26**(10).

11. Alsaleh, N., et al., *Medical and dental students' knowledge and perceptions about antimicrobial stewardship: a call for educational enhancement.* Military Medical Science Letters (Vojenske Zdravotnicke Listy), 2020. **89**(4): p. 207-214.

12. AL-Salih, S.S.H., et al., *Knowledge and attitudes regarding antibiotic use and resistance among nursing and dentistry students in Babylon University/Iraq.* Indian J Forensic Med Toxicol, 2019. **13**(4): p. 1147-52.

13. Al-Taani, G.M., et al., *Knowledge, attitude, and behavior about antimicrobial use and resistance among medical, nursing and pharmacy students in Jordan: A cross sectional study.* Antibiotics, 2022. **11**(11): p. 1559.

14. Anandakumar, S. and R. Sankari, *Knowledge, attitude and practice on antibiotic therapy among dental students-a pilot study.* Research Journal of Pharmacy and Technology, 2018. **11**(6): p. 2473-2475.

15. Assar, A., et al., *Knowledge, attitudes, and practices of Egypt’s future physicians towards antimicrobial resistance (KAP-AMR study): a multicenter cross-sectional study.* Environmental Science and Pollution Research, 2020. **27**: p. 21292-21298.

16. Augie, B.M., et al., *Knowledge and perceptions about antibiotic resistance and prudent antibiotic prescribing among final year medical students in two African countries.* International Journal of Pharmacy Practice, 2021. **29**(5): p. 508-514.

17. Azechi, T., et al., *Evaluation of Knowledge Regarding the Use of Antibiotics among Pharmacy Undergraduates in Japan.* Journal of Microbiology & Biology Education, 2022. **23**(3): p. e00146-22.

18. Baddal, B., T.J. Lajunen, and M.J. Sullman, *Knowledge, attitudes and behaviours regarding antibiotics use among Cypriot university students: a multi-disciplinary survey.* BMC Medical Education, 2022. **22**(1): p. 847.

19. Bajalan, A., et al., *Awareness regarding antimicrobial resistance and confidence to prescribe antibiotics in dentistry: a cross-continental student survey.* Antimicrobial Resistance & Infection Control, 2022. **11**(1): p. 158.

20. Bouchoucha, S.L., et al., *Nursing students’ awareness and perceptions of nurses’ role in antimicrobial stewardship.* Nurse Education in Practice, 2021. **52**: p. 103036.

21. Burger, M., et al., *Knowledge and perceptions of antimicrobial stewardship concepts among final year pharmacy students in pharmacy schools across South Africa.* Southern African Journal of Infectious Diseases, 2016. **31**(3): p. 27-33.

22. Chakravarty, A., et al., *A Multicentric Survey of Indian Medical Students about their Knowledge and Perception on Antimicrobial Stewardship.* Journal of Pure & Applied Microbiology, 2022. **16**(2).

23. Chapot, L., et al., *Knowledge, attitudes and practices regarding antibiotic use and resistance among veterinary students in Bangladesh.* Antibiotics, 2021. **10**(3): p. 332.

24. Chen, J., et al., *Lack of antibiotic knowledge and misuse of antibiotics by medical students in Mali: a cross-sectional study.* Expert Review of Anti-infective Therapy, 2021. **19**(6): p. 797-804.

25. Chuenchom, N., et al., *Perception, attitude, and knowledge regarding antimicrobial resistance, appropriate antimicrobial use, and infection control among future medical practitioners: a multicenter study.* infection control & hospital epidemiology, 2016. **37**(5): p. 603-605.

26. Doshi, A., et al., *Knowledge and practices of Indian dental students regarding the prescription of antibiotics and analgesics.* Clujul Medical, 2017. **90**(4): p. 431.

27. Dutt, H.K., et al., *A comparative knowledge, attitude, and practice study of antimicrobial use, self-medication and antimicrobial resistance among final year students of MBBS, BDS, and BSc Nursing at a tertiary care hospital at Kannur.* National Journal of Physiology, Pharmacy and Pharmacology, 2018. **8**(9): p. 1305-1305.

28. Dyar, O.J., et al., *Assessing the Knowledge, Attitudes and Behaviors of Human and Animal Health Students towards Antibiotic Use and Resistance: A Pilot Cross-Sectional Study in the UK.* Antibiotics (Basel), 2018. **7**(1).

29. Dyar, O., et al., *Knowledge, attitudes, and beliefs of French medical students about antibiotic prescribing and resistance.* Médecine et maladies infectieuses, 2013. **43**(10): p. 423-430.

30. Dyar, O.J., et al., *Preparedness to prescribe antibiotics responsibly: a comparison between final year medical students in France and Sweden.* European Journal of Clinical Microbiology & Infectious Diseases, 2019. **38**: p. 711-717.

31. Dyar, O.J., et al., *Do medical students feel prepared to prescribe antibiotics responsibly? Results from a cross-sectional survey in 29 European countries.* Journal of Antimicrobial Chemotherapy, 2018. **73**(8): p. 2236-2242.

32. Dyar, O.J., G. Tebano, and C. Pulcini, *Managing responsible antimicrobial use: perspectives across the healthcare system.* Clinical Microbiology and Infection, 2017. **23**(7): p. 441-447.

33. Dyar, O., et al., *O051: European medical students and antibiotic stewardship: a multicentre survey of knowledge, attitudes and beliefs.* Antimicrobial Resistance and Infection Control, 2013. **2**: p. 1-1.

34. Fasina, F.O., et al., *Knowledge, attitudes, and perceptions associated with antimicrobial stewardship among veterinary students: A multi-country survey from Nigeria, South Africa, and Sudan.* Frontiers in public health, 2020. **8**: p. 517964.

35. Fejza, A., et al., *Pharmacy students’ knowledge and attitudes about antibiotics in Kosovo.* Pharmacy Practice (Granada), 2016. **14**(1): p. 0-0.

36. Ferdoush, J., et al., *Knowledge, perception and preparedness of future prescribers about antimicrobial stewardship.* ||| Bangladesh Journal of Pharmacology, 2016. **11**(4): p. 928-934.

37. Golding, S.E., H.M. Higgins, and J. Ogden, *Assessing Knowledge, Beliefs, and Behaviors around Antibiotic Usage and Antibiotic Resistance among UK Veterinary Students: A Multi-Site, Cross-Sectional Survey.* Antibiotics, 2022. **11**(2): p. 256.

38. Hagiya, H., et al., *Antibiotic literacy among Japanese medical students.* Journal of Infection and Chemotherapy, 2020. **26**(10): p. 1107-1109.

39. Hanna, L.-A., C. McMichael, and M. Hall, *What do future pharmacists know about, and think of, antimicrobial stewardship?* Pharmacy Education, 2019. **19**: p. 146-154.

40. Hanweet, D.M., K.A. Mahdi, and A.Q. Ahmed, *Knowledge and Attitude of Antibiotic Prescription Among Dental Students in Najaf City, Iraq.* International Neurourology Journal, 2023. **27**(4): p. 1387-1391.

41. Haque, M., et al., *A cross-sectional study evaluating the knowledge and beliefs about, and the use of antibiotics amongst Malaysian university students.* Expert review of anti-infective therapy, 2019. **17**(4): p. 275-284.

42. Haque, M., et al., *Antibiotic prescribing and resistance: knowledge level of medical students of clinical years of University Sultan Zainal Abidin, Malaysia.* Therapeutics and clinical risk management, 2016: p. 413-426.

43. Haque, M., et al., *Antimicrobial prescribing confidence and knowledge regarding drug resistance: Perception of medical students in malaysia and the implications.* Antibiotics, 2022. **11**(5): p. 540.

44. Haque, M., et al., *Antibiotic use: A cross-sectional study evaluating the understanding, usage and perspectives of medical students and pathfinders of a public defence university in Malaysia.* Antibiotics, 2019. **8**(3): p. 154.

45. Hardefeldt, L., et al., *Veterinary students’ knowledge and perceptions about antimicrobial stewardship and biosecurity—a national survey.* Antibiotics, 2018. **7**(2): p. 34.

46. Hayat, K., et al., *Understanding of future prescribers about antimicrobial resistance and their preparedness towards antimicrobial stewardship activities in Pakistan: Findings and implications.* Frontiers in Pharmacology, 2022. **13**: p. 771083.

47. Hayat, K., et al., *Understanding of pharmacy students towards antibiotic use, antibiotic resistance and antibiotic stewardship programs: a cross-sectional study from Punjab, Pakistan.* Antibiotics, 2021. **10**(1): p. 66.

48. Higuita-Gutiérrez, L.F., et al., *Knowledge regarding antibiotic use among students of three medical schools in Medellin, Colombia: a cross-sectional study.* BMC Medical Education, 2020. **20**: p. 1-9.

49. Higuita-Gutiérrez, L.F., G.E. Roncancio Villamil, and J.N. Jiménez Quiceno, *Knowledge, attitude, and practice regarding antibiotic use and resistance among medical students in Colombia: A cross-sectional descriptive study.* BMC public health, 2020. **20**: p. 1-12.

50. Holz, M., et al., *Antibiotics and antimicrobial resistance: Evaluation of the knowledge, attitude, and perception among students and faculty within US dental schools.* Journal of Dental Education, 2021. **85**(3): p. 383-391.

51. Hoque, R., A. Mostafa, and M. Haque, *Insight of medical students of clinical years to antimicrobials prescribing and resistance in private medical school, Chittagong, Bangladesh.* Journal of Young Pharmacists, 2016. **8**(4): p. 447.

52. Horvat, O., et al., *Survey of the knowledge, attitudes and practice towards antibiotic use among prospective antibiotic prescribers in Serbia.* Antibiotics, 2022. **11**(8): p. 1084.

53. Horvat, O., et al., *Serbian students’ knowledge, attitudes and behaviour towards antibiotic use: is there room for improvement?* International journal of public health, 2020. **65**: p. 1257-1267.

54. Huang, Y., et al., *Knowledge, attitude and practice of antibiotics: a questionnaire study among 2500 Chinese students.* BMC Medical Education, 2013. **13**(1): p. 163.

55. Hussain, I., et al., *Assessing Knowledge and Perception Regarding Antimicrobial Stewardship and Antimicrobial Resistance in University Students of Pakistan: Findings and Implications.* Antibiotics, 2021. **10**(7): p. 866.

56. Hussain, M., M.A. Atif, and L. Akhtar, *IMPACT OF ANTIBIOTIC STEWARDSHIP LEARNING SESSION ON KNOWLEDGE AND ATTITUDE OF UNDERGRADUATE MEDICAL STUDENTS.* KHYBER MEDICAL UNIVERSITY JOURNAL, 2021. **13**(3): p. 152-6.

57. Hu, Y., et al., *Knowledge, attitude, and practice with respect to antibiotic use among Chinese medical students: a multicentre cross-sectional study.* International journal of environmental research and public health, 2018. **15**(6): p. 1165.

58. Jain, M.R., D. Ganapathy, and V. Sivasamy, *A survey on knowledge, attitude, and perception of antibiotic resistance and usage among dental students.* Drug Invention Today, 2020. **13**(3).

59. Jaiprakash, H., et al., *Knowledge, Attitude and Practices on Antimicrobial Resistance and Usage Among Medical Students in A Malaysian Medical University.* 2020.

60. Jairoun, A., et al., *Knowledge, attitude and practice of antibiotic use among university students: a cross sectional study in UAE.* BMC public health, 2019. **19**: p. 1-8.

61. Jamshed, S.Q., et al., *Understanding of antibiotic use and resistance among final-year pharmacy and medical students: a pilot study.* The journal of infection in developing countries, 2014. **8**(06): p. 780-785.

62. Jang, W., H. Pai, and B. Kim. *Change in the perception of oral antibiotics among medical students after participating in a parenteral-to-oral conversion program for highly bioavailable antibiotics*. in *Open Forum Infectious Diseases*. 2022. Oxford University Press US.

63. Ji, W., et al., *Perceptions and preparedness of senior medical students about antimicrobial stewardship programs: are we providing adequate training to future prescribers?* Expert Review of Anti-infective Therapy, 2023. **21**(3): p. 309-315.

64. Justo, J.A., et al., *Knowledge and attitudes of doctor of pharmacy students regarding the appropriate use of antimicrobials.* Clinical Infectious Diseases, 2014. **59**(suppl_3): p. S162-S169.

65. Kamoto, A., *Knowledge, attitude and perception on antimicrobial use and antimicrobial resistance among final year medical students in the College of Medicine, Malawi.* Malawi Medical Journal, 2020. **32**(3): p. 120-123.

66. Kandasamy, G., et al., *Knowledge, attitude, perception and practice of antibiotics usage among the pharmacy students.* International journal of clinical practice, 2020. **74**(11): p. e13599.

67. Kanyike, A.M., et al., *Antimicrobial resistance and rational use of medicine: knowledge, perceptions, and training of clinical health professions students in Uganda.* Antimicrobial Resistance & Infection Control, 2022. **11**(1): p. 145.

68. Khan, F.U., et al., *Exploring Undergraduate Pharmacy Students Perspectives Towards Antibiotics Use, Antibiotic Resistance, and Antibiotic Stewardship Programs Along With the Pharmacy Teachers' Perspectives: A Mixed-Methods Study From Pakistan.* Front Pharmacol, 2021. **12**: p. 754000.

69. Indrapriyadharshini, K., et al., *Knowledge about antibiotic resistance among dental students in Chengalpattu district, Tamil Nadu – A cross-sectional study.* Journal of Global Oral Health, 2021. **4**.

70. Kovacevic, Z., et al., *Mapping knowledge and comprehension of antimicrobial stewardship and biosecurity among veterinary students.* PLoS One, 2020. **15**(8): p. e0235866.

71. Lubwama, M., et al., *Preparedness of medicine and pharmacy students in Sub-Saharan Africa to prescribe antibiotics appropriately in an era of antimicrobial resistance.* International Journal of Infectious Diseases, 2020. **101**: p. 101.

72. Lubwama, M., et al., *Knowledge, attitudes, and perceptions about antibiotic use and antimicrobial resistance among final year undergraduate medical and pharmacy students at three universities in East Africa.* PLoS One, 2021. **16**(5): p. e0251301.

73. Zhao, A., et al., *Knowledge, perception, and educational status of antimicrobial resistance among Chinese medical students.* Microbial Drug Resistance, 2019. **25**(10): p. 1458-1464.

74. Zawahir, S., C. Hettiarachchi, and H. Morrissey, *Assessing knowledge, perception and attitudes about antibiotics among final year pharmacy undergraduates in Sri Lanka.* 2017.

75. Yuste, J.R., A.B.-D. Matteo, and F. Gruber, *Impact of Infectious Diseases training in the perception of antibiotic resistance and rational use of antibiotics among Spanish medical students–a cross-sectional study.* BMC Medical Education, 2022. **22**(1): p. 550.

76. Yilmaz, Z. and Z.G. Yorguner, *Pharmacy students' knowledge and attitudes towards antibiotic use: A cross-sectional study.* Journal of Research in Pharmacy, 2023. **27**(5).

77. Yang, K., et al., *Attitudes and perceptions regarding antimicrobial use and resistance among medical students in Central China.* Springerplus, 2016. **5**: p. 1-8.

78. Yahya, R.N. and M.M. Wasmi, *A Comparative Study about Knowledge, Attitude, Practice of Antibiotic Use and Perceptions of the Possible Causes of Resistance between Final Year Undergraduate Students and Postgraduate Pharmacy Students.* Medico-legal Update, 2021. **21**(2).

79. Wright, E. and P. Jain, *Survey of antibiotic knowledge amongst final year medical students.* Journal of Antimicrobial Chemotherapy, 2004. **53**(3): p. 550-551.

80. Wong, Y.C., M. Mohan, and A. Pau, *Dental students′ compliance with antibiotic prescribing guidelines for dental infections in children.* Journal of Indian Society of Pedodontics and Preventive Dentistry, 2016. **34**(4): p. 348-353.

81. Wiese-Posselt, M., et al., *Appropriate antibiotic use and antimicrobial resistance: knowledge, attitudes and behaviour of medical students and their needs and preferences for learning.* Antimicrob Resist Infect Control, 2023. **12**(1): p. 48.

82. Weier, N., K. Thursky, and S.T.R. Zaidi, *Antimicrobial knowledge and confidence amongst final year medical students in Australia.* PloS one, 2017. **12**(8): p. e0182460.

83. Wasserman, S., et al., *South African medical students’ perceptions and knowledge about antibiotic resistance and appropriate prescribing: are we providing adequate training to future prescribers?* South African medical journal, 2017. **107**(5): p. 405-410.

84. Vo, T.Q., *KNOWLEDGE, ATTITUDE AND PRACTICE OF ANTIBIOTICS AMONG MEDICAL STUDENTS IN VIETNAM.* Eurasian Journal of Analytical Chemistry, 2018.

85. Vázquez-Lago, J.M., et al., *Knowledge, Perceptions, and Perspectives of Medical Students Regarding the Use of Antibiotics and Antibiotic Resistance: A Qualitative Research in Galicia, Spain.* Antibiotics (Basel), 2023. **12**(3).

86. Thriemer, K., et al., *Antibiotic prescribing in DR Congo: a knowledge, attitude and practice survey among medical doctors and students.* PloS one, 2013. **8**(2): p. e55495.

87. KULSOOM TAYYAB, I.S., FATIMA MUKHTAR, HASNAIN ALI SHAHID, IMRAN TAHIR, HUSSNAIN GOHAR, *Knowledge and Perceptions of Medical Students Regarding Antibiotic Use.* Pakistan Journal of Medical & Health Sciences, 2017. **11**.

88. Tariq, K., et al., *Awareness of antibiotic use and antibiotic resistance amongst dental students.* Brazilian Dental Science, 2021. **24**(3).

89. Struzycka, I., et al., *Knowledge of antibiotics and antimicrobial resistance amongst final year dental students of Polish medical schools—A cross‐sectional study.* European Journal of Dental Education, 2019. **23**(3): p. 295-303.

90. Sobierajski, T., et al., *Knowledge-based attitudes of medical students in antibiotic therapy and antibiotic resistance. A cross-sectional study.* International Journal of Environmental Research and Public Health, 2021. **18**(8): p. 3930.

91. Sobierajski, T., et al., *Antimicrobial and antibiotic resistance from the perspective of Polish veterinary students: an inter-university study.* Antibiotics, 2022. **11**(1): p. 115.

92. Smith, P.W., et al., *Implication of the knowledge and perceptions of veterinary students of antimicrobial resistance for future prescription of antimicrobials in animal health, South Africa.* Journal of the South African Veterinary Association, 2019. **90**(1): p. 1-8.

93. Shrestha, R., *Knowledge, attitude and practice on antibiotics use and its resistance among medical students in a tertiary care hospital.* JNMA: Journal of the Nepal Medical Association, 2019. **57**(216): p. 74.

94. PANDURANGAN, K.K., *Cross Sectional Survey Of Knowledge, Attitude And Practice Of Antibiotic Use Among Dental And Paramedical Students.* International Journal of Pharmaceutical Research (09752366), 2020.

95. Sharma, N., et al., *Prevalence of self-medication of antibiotics among 2nd-year medical students and their knowledge about antibiotic resistance.* International Journal of Academic Medicine, 2020. **6**(3): p. 203-208.

96. Sharma, K., P. Jain, and A. Sharma, *Knowledge, attitude and perception of medical and dental undergraduates about antimicrobial stewardship.* Indian journal of pharmacology, 2015. **47**(6): p. 676-679.

97. Arun Kumar Sharma, V.G.a.S.S., *KNOWLEDGE, ATTITUDE AND PRACTICE TOWARDS ANTIBIOTIC USE AND ANTIBIOTIC RESISTANCE AMONG MEDICAL STUDENTS: A CROSS-SECTIONAL STUDY.* International Journal of Pharmaceutical Sciences and Research, 2022. **13**.

98. Shah, S., et al., *Antibiotic use: A cross-sectional survey assessing the knowledge, attitudes, and practices amongst students of Punjab, Pakistan.* Journal of American College Health, 2022. **70**(8): p. 2499-2504.

99. Shah, P., et al., *Knowledge, attitude, and practice associated with antibiotic use among university students: a survey in Nepal.* International journal of environmental research and public health, 2019. **16**(20): p. 3996.

100. Seid, M.A. and M.S. Hussen, *Knowledge and attitude towards antimicrobial resistance among final year undergraduate paramedical students at University of Gondar, Ethiopia.* BMC infectious diseases, 2018. **18**: p. 1-8.

101. Sefah, I.A., et al., *Evaluation of healthcare students’ knowledge on antibiotic use, antimicrobial resistance and antimicrobial stewardship programs and associated factors in a tertiary university in Ghana: Findings and implications.* Antibiotics, 2022. **11**(12): p. 1679.

102. Schneider-Smith, E.G., et al., *How decisions are made: Antibiotic stewardship in dentistry.* Infect Control Hosp Epidemiol, 2023. **44**(11): p. 1731-1736.

103. Scaioli, G., et al., *Antibiotic use: a cross-sectional survey assessing the knowledge, attitudes and practices amongst students of a school of medicine in Italy.* PloS one, 2015. **10**(4): p. e0122476.

104. Bp, S., et al., *Survey on knowledge towards antibiotics among the nursing students.* International journal of Pharmacy and Pharmaceutical Sciences, 2011. **3**.

105. Sarnino, N., et al., *Evaluation of the education in antimicrobial stewardship and resistance by students of two Belgian Faculties of Veterinary Medicine.* Vlaams Diergeneeskundig Tijdschrift, 2022. **91**(5).

106. Sannathimmappa, M.B., V. Nambiar, and R. Aravindakshan, *A cross-sectional study to evaluate the knowledge and attitude of medical students concerning antibiotic usage and antimicrobial resistance.* International Journal of Academic Medicine, 2021. **7**(2): p. 113-119.

107. Sánchez-Fabra, D., et al., *Perspective of Spanish medical students regarding undergraduate education in infectious diseases, bacterial resistance and antibiotic use.* Enfermedades infecciosas y microbiologia clinica (English ed.), 2019. **37**(1): p. 25-30.

108. Saleem, Z., et al., *Medical and pharmacy students’ knowledge, attitude and perception concerning antimicrobial use and resistance in Pakistan.* Pharmacy Education, 2019. **19**: p. 199-205.

109. Sakeena, M.H., et al., *Investigating knowledge regarding antibiotics among pharmacy and allied health sciences students in a Sri Lankan university.* The Journal of Infection in Developing Countries, 2018. **12**(09): p. 726-732.

110. Sakeena, M., A.A. Bennett, and A.J. McLachlan, *Investigating knowledge regarding antibiotics and antimicrobial resistance among pharmacy students in Australian universities.* Journal of Pharmacy Practice and Research, 2021. **51**(1): p. 54-61.

111. Sakeena, M., et al., *A comparative study regarding antibiotic consumption and knowledge of antimicrobial resistance among pharmacy students in Australia and Sri Lanka.* PLoS One, 2019. **14**(3): p. e0213520.

112. Rusic, D., et al., *Attitudes and knowledge regarding antimicrobial use and resistance among pharmacy and medical students at the University of Split, Croatia.* Microbial drug resistance, 2018. **24**(10): p. 1521-1528.

113. Ritchie, O., et al., *Confidence in antibiotic prescribing intentions among senior medical students in India.* The American Journal of Tropical Medicine and Hygiene, 2020. **103**(6): p. 2561.

114. Reena, A.P. and A.M. Ittyachen, *Awareness of antibiotic resistance among medical students in Kerala State, India: A cross-sectional study.* Current Medical Issues, 2022. **20**(4): p. 245-252.

115. Rattanaumpawan, P., N. Chuenchom, and V. Thamlikitkul, *Perception, attitude, knowledge and learning style preference on challenges of antimicrobial resistance and antimicrobial overuse among first year doctors in training and final year medical students.* Antimicrobial Resistance & Infection Control, 2019. **8**: p. 1-7.

116. Rajni, E., et al., *Impact of Hospital Acquired Infection and Antibiotic Resistance Awareness Campaign on Knowledge Attitude and Practices of Medical Undergraduates in a Tertiary Care Teaching Hospital, India.* J Clin Diagn Res, 2020. **14**: p. 23-27.

117. Rajiah, K., W.S. Ren, and S.Q. Jamshed, *Evaluation of the understanding of antibiotic resistance among Malaysian pharmacy students at public universities: an exploratory study.* Journal of infection and public health, 2015. **8**(3): p. 266-273.

118. Raees, I., et al., *Understanding of final year medical, pharmacy and nursing students in Pakistan towards antibiotic use, antimicrobial resistance and stewardship: findings and implications.* Antibiotics, 2023. **12**(1): p. 135.

119. Rábano-Blanco, A., et al., *Nursing Students' Knowledge and Awareness of Antibiotic Use, Resistance and Stewardship: A Descriptive Cross-Sectional Study.* Antibiotics (Basel), 2019. **8**(4).

120. Primeau, C.A., et al., *Exploring medical and veterinary student perceptions and communication preferences related to antimicrobial resistance in Ontario, Canada using qualitative methods.* BMC Public Health, 2023. **23**(1): p. 483.

121. Mariona, P. and T. Lakshmi, *Survey on knowledge, attitude and practice of antimicrobial resistance among dental students.* Int J Pharm Sci Rev Res, 2017. **43**(2): p. 5-7.

122. Popoola, O.O., et al., *The knowledge and perception of pharmacy students and recent graduates in IPSF toward antimicrobial stewardship.* Pharmacy Education, 2022. **22**(1): p. 682-687.

123. Phagava, H., et al., *SURVEY OF PRACTICES, KNOWLEDGE AND ATTITUDE CONCERNING ANTIBIOTICS AND ANTIMICROBIAL RESISTANCE AMONG MEDICAL UNIVERSITY STUDENTS.* Georgian Medical News, 2019(294): p. 77-82.

124. Padmanabha, T. and Y. Rajashekar, *Emerging causes for antibiotic resistance: second-year medical undergraduate’s perspective: a cross-sectional study.* 2018.

125. Owusu-Ofori, A.K., et al., *Self-medication and antimicrobial resistance: a survey of students studying healthcare programmes at a tertiary institution in Ghana.* Frontiers in public health, 2021. **9**: p. 706290.

126. Ortiz-Martínez, Y. and C. Moreno-Babilonia, *Antibiotic prescribing and resistance: knowledge among Latin American medical students during Antibiotic Awareness Week 2016.* Journal of Hospital Infection, 2017. **96**(3): p. 301.

127. Olorukooba, A.A., et al., *Antimicrobial stewardship: Perception and familiarity of future prescribers in a tertiary health institution in Northern Nigeria.* Sahel Medical Journal, 2020. **23**(4): p. 236-241.

128. Alex, I.O., *Knowledge of antibiotic use and resistance among students of a medical school in Nigeria.* Malawi Medical Journal, 2019. **31**(2): p. 133-137.

129. Odetokun, I.A., et al., *Knowledge of antimicrobial resistance among veterinary students and their personal antibiotic use practices: A national cross-sectional survey.* Antibiotics, 2019. **8**(4): p. 243.

130. Nowbuth, A.A., et al., *Assessment of the knowledge, attitudes and perceived quality of education about antimicrobial use and resistance of medical students in Zambia, Southern Africa.* JAC-Antimicrobial Resistance, 2023. **5**(3): p. dlad073.

131. Nori, P., et al. *Faces of resistance: using real-world patients and their advocates to teach medical students about antimicrobial stewardship*. in *Open Forum Infectious Diseases*. 2019. Oxford University Press US.

132. Nisabwe, L., et al., *Knowledge and attitudes towards antibiotic use and resistance among undergraduate healthcare students at University of Rwanda.* Journal of Pharmaceutical Policy and Practice, 2020. **13**(1): p. 7.

133. Mubarak, N., et al., *How Are We Educating Future Physicians and Pharmacists in Pakistan? A Survey of the Medical and Pharmacy Student’s Perception on Learning and Preparedness to Assume Future Roles in Antibiotic Use and Resistance.* Antibiotics, 2021. **10**(10): p. 1204.

134. Minen, M.T., et al., *A survey of knowledge, attitudes, and beliefs of medical students concerning antimicrobial use and resistance.* Microb Drug Resist, 2010. **16**(4): p. 285-9.

135. Min, S., et al., *Knowledge, attitude, and practice associated with antimicrobial resistance among medical students between 2017 and 2022: a survey in East China.* Frontiers in Public Health, 2022. **10**: p. 1010582.

136. Mikhael, E.M., M.K. Hasan, and S.Z. Abdulridha, *Assessment of antibiotic knowledge among final year pharmacy students at University of Baghdad.* The Open Public Health Journal, 2019. **12**(1).

137. Meher, B.R., et al., *Factors most influencing antibiotic stewardship program and comparison of prefinal-and final-year undergraduate medical students.* Perspectives in Clinical Research, 2020. **11**(1): p. 18-23.

138. Mate, V.H., et al., *Knowledge, attitude and practice towards antibiotic use: An interventional study among medical and dental undergraduates.* National Journal of Physiology, Pharmacy and Pharmacology, 2022. **12**(3): p. 350-350.

139. Marta-Costa, A., et al., *Survey of the Knowledge and Use of Antibiotics among Medical and Veterinary Health Professionals and Students in Portugal.* International Journal of Environmental Research and Public Health, 2021. **18**(5): p. 2753.

140. Priyesh Marskole, V.S.C., Satish Chandel, Ritesh Churihar, *Assessment of knowledge, attitudes, and practices about antibiotic resistance among medical students in Central India.* European Journal of Molecular & Clinical Medicine, 2022. **9**(6).

141. Manikanta, K.N., et al., *Antibiotic use and resistance: A cross-sectional study exploring knowledge, attitudes and practices among medical, dental, pharmacy and nursing students.* Journal of Pharmaceutical Negative Results, 2022: p. 3543-3558.

142. Nitesh Mangal, M.B., Jitendra Kumar, Chintan Doshi, *KAP on Antibiotic Usage and Resistance among Second Professional Medical Students.* International Journal of Pharmaceutical and Clinical Research, 2023. **15**(2).

143. Malli, I.A., M.S. Mohamud, and S. Al-Nasser, *Enhancing Medical Students’ Confidence and Knowledge in Antibiotic Prescription and Administration through Virtual Education: A Quasi-Experimental Study.* Antibiotics, 2023. **12**(10): p. 1546.

144. MacDougall, C., et al., *An Interprofessional Curriculum on Antimicrobial Stewardship Improves Knowledge and Attitudes Toward Appropriate Antimicrobial Use and Collaboration.* Open Forum Infectious Diseases, 2017. **4**(1).
